# Human milk microbial species are associated with mild growth deficits during early infancy among Guatemalan mother–infant dyads

**DOI:** 10.3389/frmbi.2022.1008467

**Published:** 2022-11-25

**Authors:** Tamara T. Ajeeb, Emmanuel Gonzalez, Noel W. Solomons, Kristine G. Koski

**Affiliations:** ^1^ School of Human Nutrition, McGill University, Montreal, QC, Canada; ^2^ Department of Clinical Nutrition, College of Applied Medical Sciences, Umm Al-Qura University, Makkah, Saudi Arabia; ^3^ Canadian Centre for Computational Genomics, McGill Genome Centre, Montréal, QC, Canada; ^4^ Department of Human Genetics, McGill University, Montréal, Montréal, QC, Canada; ^5^ Gerald Bronfman Department of Oncology, McGill University, Montréal, QC, Canada; ^6^ Center for Studies of Sensory Impairment, Aging and Metabolism (CeSSIAM), Guatemala City, Guatemala

**Keywords:** human milk microbiome, lactation, infant growth, z-scores, WAZ and LAZ, Guatemala, Indigenous mothers, 16S rRNA gene

## Abstract

Growth faltering is common in Guatemalan indigenous communities, but the possibility that it may be related to milk microbial composition has not been explored. For this cross-sectional study, unrelated mother–infant dyads (*n* = 64) from eight communities in the remote Western Highlands of Guatemala were recruited. Milk samples and infant length-for-age and weight-for-age *Z*-scores were collected at two stages of lactation: early (6–46 days postpartum, *n* = 29) or late (109–184 days postpartum, *n* = 35). Within each stage of lactation, infants were subdivided into mildly underweight [weight-for-age *Z*-score (WAZ) < –1 SD] or normal weight (WAZ ≥ –1 SD) and mildly stunted [length-for-age *Z*-score (LAZ) < –1.5 SD] or non-stunted (LAZ ≥ –1.5 SD). 16S ribosomal RNA gene sequencing was used to identify milk microbial communities, and DESeq2 was used to compare the differential abundance (DA) of human milk microbiota at the species level for WAZ and LAZ subgroups at each stage of lactation. A total of 503 ESVs annotated as 256 putative species across the 64 human milk samples were identified. Alpha diversity did not differ, but beta-diversity redundancy analysis identified four distinct clusters among the four WAZ (*p* = 0.004) and LAZ subgroups (*p* = 0.001). DA identified 15 different taxa in the WAZ and 11 in the LAZ groups in early lactation and 8 in the WAZ and 19 in the LAZ groups in late lactation. Mothers’ milk had more DA taxa of oropharyngeal and environmental bacteria with opportunistic activities in the LAZ < –1.5 SD infants, whereas the LAZ ≥ –1.5 SD had DA taxa with potential probiotic and antimicrobial inhibitory activity against pathogens. In particular, milk microbial communities of infants not classified as underweight or stunted had more beneficial species including *Lactococcus_lactis*. These findings suggest the potential associations between the milk microbiome at the species level with infant growth prior to 6 months of age. These data provide important evidence of the associations between the human milk microbiome and the growth of breastfed infants.

## Introduction

In 2020, the UNICEF reported that more than one in five children under 5 years old or 149 million were stunted, and 45 million suffered from wasting; these children were largely from low- to middle-income countries, where exclusive breastfeeding predominates (UNICEF/WHO/World Bank, 2020). In Guatemala, a developing country with high exclusive breastfeeding rates (76.8%) (Colombara et al., 2015), more than 90% of indigenous mothers will breastfeed beyond 6 months (Brown et al., 2016). Nonetheless, Guatemala has the highest prevalence of stunting in the Americas, with Mayan indigenous communities disproportionately affected (World Bank, 2021). Although maternal malnutrition can contribute to growth faltering during gestation (Solomons et al., 2015), *in-utero* growth restriction does not fully explain stunting during the first few months after birth among exclusively breastfed infants given earlier reports that growth faltering starts soon after birth in rural Guatemala (Ruel et al., 1995) and is present by 3–6 months even among breastfed Guatemalan infants (Rivera and Ruel, 1997).

Human milk is known to promote optimum growth of the infant through diverse nutritional and non-nutritional compositions (Geddes et al., 2021; Perrella et al., 2021). However, there is accumulating evidence to suggest that the suboptimal nutrient composition of breast milk, especially among malnourished mothers in developing countries (Dror and Allen, 2018; Erick, 2018; Daniels et al., 2019), might underscore infant growth faltering in breastfed infants during early lactation. Researchers have reported that human milk lactose concentrations at 2 weeks and 4 months postpartum were positively associated with infant WLZ trajectories to 6 months of age (Young et al., 2017); that daily intake of human milk carbohydrates was related to infant anthropometry and body composition during the first year (Gridneva et al., 2019); that lower protein content in human milk compared with cow’s milk formulas resulted in lower lean mass and slower weight gain trajectory (Luque et al., 2015); that total lipid content of human milk has been linked to lower infant weight, adiposity, and BMI gain between 3 and 12 months (George et al., 2020); and that energy and macronutrient (carbohydrate, protein, fat) intake was associated with weight-for-length *Z*-scores at 3 months of age (Davis et al., 2019). In developing countries, recent evidence has demonstrated that maternal undernutrition during lactation is associated with lower breast milk micronutrient concentrations (Dror and Allen, 2018; Erick, 2018; Daniels et al., 2019). In India, low milk B_12_ was associated with failure-to-thrive in infants between 1.5 and 9 months of age (Akcaboy et al., 2015), whereas concentrations at 6 months were not correlated with Kenyan infant length or weight (Neumann et al., 2013). Among Guatemalan mother–infant dyads during the first month of birth, infant weight-for-age *Z*-score (WAZ) and length-for-age *Z*-score (LAZ) were positively associated in breastfed infants using estimated intakes calculated from milk concentrations of calcium, magnesium, potassium, rubidium, and strontium (using the principal component analysis) and, additionally, to sodium at 4–6 months of age (Li et al., 2016). More recently, infant linear growth velocity before 46 days postpartum was associated with milk magnesium intake (Li et al., 2019).

Human milk also supplies a continuous source of microbiota and is responsible for seeding the infant’s gut (Fitzstevens et al., 2017; Perrella et al., 2021; Stinson et al., 2021). This milk microbiome contributes to shaping infant oral and gut microbiomes (Stewart et al., 2018); aids in nutrient digestion (Turroni et al., 2018), energy harvesting, and energy storage (Pannaraj et al., 2017); and may play an overlooked role in infant growth (Arrieta et al., 2014; Geddes et al., 2021; Perrella et al., 2021). Studies have reported that intestinal microbiota affects postnatal growth kinetics despite chronic undernutrition (Schwarzer et al., 2016; Schwarzer et al., 2018). Others have observed associations between infant gut microbiota and infant growth (Gough et al., 2015; Davis et al., 2017; Pastor-Villaescusa et al., 2020; Carney et al., 2021). In a case–control analysis of a twin cohort of Malawian and Bangladeshi infants, linear growth deficits were inversely correlated with the relative abundance of *Acidaminococcus* sp. (Gough et al., 2015) and that infant WAZ during the first year of life was associated with infant gut microbiome phyla; *Ascomycota*, *Basidiomycota*, and *Planctomycetes* were positively associated, whereas *Cyanobacteria* and *Synergistetes* were negatively associated (Carney et al., 2021). A few studies have explored the associations between infant growth parameters, infant gut microbiota, and human milk oligosaccharide (HMO) composition reporting associations with infant growth; *Bacilli* with infant stunting (HAZ < −2) and *Adlercreutzia* and *Klebsiella* were enriched in wasted infants (WAZ < −2) at 20 weeks postpartum (Davis et al., 2017) and significant associations between HMO composition and height and weight *Z*-scores between 3 and 12 months (Lagström et al., 2020). HMOs also serve as metabolic substrates for the growth of beneficial bacteria (Lyons et al., 2020) and the prevention of pathogenic bacteria colonization through anti-adhesive properties in the infant’s intestine (Wiciński et al., 2020).

None of these previous studies had investigated the associations of the human milk microbiome (HMM) with infant growth faltering in exclusively breastfed infants prior to 6 months of age. Thus, the purpose of this study was to explore the association between growth faltering during early lactation and the HMM at the species level among lactating mother–infant dyads living in the Western Highlands of Guatemala. To minimize the exchange of microbes, mothers were recruited from eight distinct remote communities. Our specific aims for this cross-sectional study were to identify a possible association of the HMM with growth faltering at early (6–46 days postpartum) and late (109–184 days postpartum) in the first 6 months of lactation. Infants were categorized based on WAZ [normal weight (WAZ ≥ –1 SD) and mildly underweight (WAZ < –1 SD)] and LAZ [non-stunted (LAZ ≥ –1.5 SD) and mildly stunted (LAZ < –1.5 SD)].

## Methods

### Study setting, recruitment, and ethics

This study was conducted in eight rural *Mam*-Mayan communities in the Western Highlands of Guatemala between June 2012 and January 2013 and was a collaboration between McGill University and the Center for Studies of Sensory Impairment, Aging, and Metabolism (CeSSIAM), a research organization based in Guatemala. Lactating mothers were recruited by community health workers using a participatory action research framework (Cargo and Mercer, 2008) *via* home visits, loudspeaker announcements, and word-of-mouth invitations. Ethical approvals were obtained from the ethics boards at McGill University and at CeSSIAM. Further approvals were obtained from community leaders and the local authorities of the Ministry of Health. Mothers signed or thumb-printed the written consent form if they wished to participate, and all mothers were informed of their rights to withdraw from the study at any time (Chomat et al., 2015). Mothers in the *Mam-*Mayan communities comply with the WHO recommendations to exclusively breastfeed for the first 6 months after birth (Wren et al., 2015; World Health Organization, 2017).

### Study design

For this cross-sectional study, length and weight *Z*-scores of vaginally delivered breastfed infants were collected from 64 unrelated *Mam*-Mayan mothers at two stages of lactation: early (6–46 days postpartum) or late (109–184 days postpartum). Infants were further subdivided based on the classification of weight and length *Z*-scores at each stage of lactation using the following criteria: mildly stunted [LAZ < –1.5 SD (early: *n* = 18; late: *n* = 19)] vs. non-stunted [LAZ ≥ –1.5 SD (early: *n* = 11; late: *n* = 16)] and mildly underweight [WAZ < –1 SD (early: *n* = 9; late: *n* = 15)] vs. normal weight [WAZ ≥ –1 SD (early: *n* = 20 late: *n* = 20)]. The inclusion criteria were healthy mother–infant dyads aged 6 to 46 days and 109 to 184 days postpartum and mothers who delivered vaginally and breastfed their infants exclusively or predominantly (added *aguitas*, a ritual fluid) for 6 months. The exclusion criteria were non-singleton births, infants younger than 4 days due to the possibility of still providing colostrum, insufficient milk volumes for analysis, antibiotic treatments, and mothers with subclinical mastitis (milk Na : K > 0.6) due to the possible effect of subclinical inflammation on milk microbiome community and infant growth (Wren et al., 2015; Li et al., 2019).

### Human milk sample collection

Prior to milk sample collection, a trained midwife cleaned the nipple and areola of the breast not recently used for breastfeeding with 70% ethanol. Milk sample collection was conducted *via* full manual expression by the trained midwife in a 3-h time window between 9 a.m. and 12 p.m. (Wren et al., 2015). Milk samples were collected into sterile 60-ml plastic vials and stored on ice immediately. Thereafter, in the field laboratory, milk samples were partitioned into four 15-ml vials and were stored at −30°C before being shipped to McGill University in two separate shipments (Li et al., 2016).

### Infant anthropometry

Two trained Guatemalan nutritionists took infant anthropometric measurements according to standardized procedures. The detailed methodology was previously described and published (Chomat et al., 2015). In brief, infant anthropometric measurements—length, weight, and head circumference—were measured thrice, and the mean of the three measurements was considered the final value. Infant length (cm) was measured in a recumbent supine position using an infantometer, a mobile baby measuring mat (SECA 210), and recorded to the nearest 0.5 cm. Infant weight (kg) was measured using a digital infant scale (SECA 354) and rounded to the nearest 100 g. Infant anthropometric measurements and their mother’s milk samples were collected on the same day. Infant age was calculated from the date of birth recorded on the maternal health card, or in the absence of the health card, infant age was obtained from the mother. Infant growth status indicators including length-for-age *Z*-score (LAZ) and weight-for-age Z-score (WAZ) were calculated using the World Health Organization Anthro software (3.1) (World Health Organization, 2006).

Stunting at birth and during the first month of life has been reported among Guatemalan infants (median age 19 days) where the median rural HAZ was –1.56 SD (Solomons et al., 2015). In 141 developing countries, mild stunting (i.e., < –1 SD) was common with a mean HAZ of –1.16 SD and ranged between −1.29 and −1.04 (Stevens et al., 2012). Thus, we used LAZ < –1.5 SD to define mild stunting instead of LAZ < –1 SD. Infants were subdivided by infant LAZ into non-stunted (LAZ ≥ –1.5 SD) and the mildly stunted (LAZ < –1.5 SD) and by infant WAZ into normal weight (WAZ ≥ –1 SD) and mildly underweight (WAZ < –1 SD) at both stages of lactation.

### 16S rRNA gene amplification and sequencing

DNA extraction from 1 ml of milk was performed using the DNeasy Blood and Tissue mini kit from Qiagen according to the manufacturer’s protocol by Genome Quebec. The universal eubacteria primers 27F/533R (27F: AGAGTTTGATCCTGGCTCAG, 533R: TTACCGCGGCTGCTGGCAC) were used for PCR amplification of the variable regions V1–V3 consisting of ~526 bp based on the *Escherichia coli* 16S rRNA gene (Cabrera-Rubio et al., 2012; Mediano et al., 2017; Lackey et al., 2019). The primers were chosen due to their high coverage of most genera currently considered “core” in human milk, including the genus *Cutibacterium* (Hunt et al., 2011; Jost et al., 2013). Amplification was conducted at Genome Quebec at McGill University, and sequencing was performed using Illumina MiSeq. Reagent controls were below the detection limit used by Genome Quebec for quality assurance. These amplification conditions have been previously described (Gonzalez et al., 2021).

### Microbial data processing

In this study, the ANCHOR pipeline was used to process amplicon sequences. ANCHOR is a platform that uses direct paired-end sequences, which helps to substantially improve the sequence resolution of 16S rRNA gene amplification data (Gonzalez et al., 2019). Thus, it is designed for improved species-level microbial identification. Furthermore, it uses integrated multiple-reference database annotation to enhance the interpretation of complex, non-reference microbiomes (Gonzalez et al., 2019). Mothur was used to align and dereplicate sequences (Schloss et al., 2009). Multiple databases, namely, NCBI 16S rRNA RefSeq (O'Leary et al., 2016), NCBI non-redundant nucleotide (https://www.ncbi.nlm.nih.gov), SILVA (Pruesse et al., 2007), and the Ribosomal Database Project (RDP) (Maidak et al., 2000), were used to annotate exact sequence variants (ESVs) using BLASTn with the criteria of >99% for identity and coverage. The term ESV was used here given the high criteria used (>99%) for identity and coverage providing high confidence and resolution, which maximizes biological discovery (Gonzalez et al., 2019). Priority was given to NCBI 16S rRNA RefSeq, when 100% identity and coverage hits returned across multiple databases, due to the high standard of curation. Low-count (<36) amplicons were binned to high-count ESVs at a lower threshold of >98% identity/coverage. As a caution and due to database errors, taxonomy annotation, particularly species calls, should be considered putative even when sharing 100% sequence identity to a single species.

Contamination controls were performed at multiple steps of this analysis. First, a trained midwife followed the aseptic sampling protocol which included cleaning the nipple and areola of the breast with 70% ethanol prior to milk sample collection. Second, Genome Quebec followed the aseptic technique while performing the PCR blanks. In this step, samples with visible bands in the negative controls were not sequenced. Third, the Canadian Centre for Computational Genomics (C3G) of McGill University utilized contamination controls *via* sample preprocessing while performing the bioinformatics analyses including controlling for prevalence and sparsity, ordination analysis, and identifying putative contamination (*Decontam*, R package). Putative contamination was flagged using *Decontam*. Only one OTU out of 1505 was flagged as potential contamination, although it was not selected by DESeq2 as differentially abundant (**Supplementary File 1**).

### Bioinformatics

To address sparsity issues, ESVs had to have at least three counts in three different samples from the same condition of comparison. Alpha diversity was performed without normalization. However, for beta-diversity analysis, rlog normalization (rlog function in Phyloseq R package) was used for data transformation (Gonzalez et al., 2021). Six alpha-diversity metrics were performed (Observed, Chao-1, Shannon, Simpson, Inverse Simpson, and Fisher) using *t*-tests (parametric) or Mann–Whitney *U* (non-parametric) using *Phyloseq* R package with R Studio software (version 1.4.1106) (McMurdie and Holmes, 2013) to estimate and compare microbial richness within samples. Comparing microbial richness between the LAZ infant groups and between the WAZ infant groups was done using *t*-tests on the richness measures. ACE and Chao-1 were used to account for taxonomies that were undetected due to low abundance. We used Observed to calculate the total number of unique ESVs per sample. To account for equitability in sample distribution, the Shannon index was used, and for the species dominance, we used Simpson. Fisher was used to account for uncertainty in richness estimations.

Beta diversity was used to determine and evaluate differences in HMM communities between the mildly stunted (LAZ < –1.5 SD) vs. the non-stunted (LAZ ≥ –1.5 SD) groups and between the mildly underweight (WAZ < –1 SD) vs. the normal weight (WAZ ≥  –1 SD) at both early and late lactation. To evaluate and visualize the differences between the different groups, constrained ordination was employed using rlog-transformed counts with redundancy (RDA) analysis.

Lastly, the DESeq2 procedure (Love et al., 2014) was used to evaluate differentially abundant (DA) taxa between the mildly stunted (LAZ < –1.5 SD) and the non-stunted (LAZ ≥ –1.5 SD) and between the mildly underweight (WAZ < –1 SD) and the normal weight (WAZ ≥ –1 SD) groups at both early and late lactation in order to identify statistical differences between microbial communities. DESeq2 identifies significant differences between groups while considering the library size. Differences in abundance between microbial communities with a false discovery rate (FDR < 0.05) were considered significant. Due to the exploratory nature of this study, DA ESVs with low abundances were considered.

## Results

### Characterization of Guatemalan mother–infant dyads

**Table 1** describes the maternal and infant characteristics by stage of lactation. In both early and late lactation, maternal characteristics did not differ among subgroups for age, height, weight, BMI, parity, or breastfeeding practices with the exception of Na:K ratio but only in early lactation (*p* = 0.021). Furthermore, in early lactation, no differences in head circumference were observed, but infants classified as mildly stunted (LAZ < –1.5 SD) had lower weight for age *Z*-scores (−1.56 vs. −0.35, *p* < 0.0001) in early lactation. In late lactation, mild stunting was more prevalent among male infants (63%) (*p* = 0.024) and was associated with a lower weight-for-age (*p* < 0.0001) and lower head circumference-for-age *Z*-scores (*p* < 0.0001). Moreover, infants who were classified as underweight with a WAZ < –1 SD also had lower LAZ (−2.82 vs. −1.21, *p* = 0.0001) and smaller head circumference-for-age *Z*-scores (−1.85 vs. −0.051, *p* = 0.0013) at 4–6 months postpartum.

**Table 1:** Characteristics of Guatemalan infants and mothers at two lactation stages.

	Early lactation, mean ± SD or %^a^				Late lactation, mean ± SD or %^a^			
	WAZ ≥ −1SD	WAZ < −1SD	LAZ ≥ −1.5SD1	LAZ < −1.5SD	WAZ ≥ −1SD	WAZ < −1SD	LAZ ≥ −1.5SD	LAZ < −1.5SD
*N*	20	9	11	18	20	15	16	19
**Maternal characteristics**								
Age, years	23 ± 5	23 ± 6	23 ± 6	24 ± 5	23 ± 6	25 ± 7	25 ± 6	23 ± 7
Height, cm	147.4 ± 4	144.2 ± 6	147.5 ± 4.1	145.7 ± 5.3	148.5 ± 5.4	146.6 ± 4.7	148.6 ± 5.8	147 ± 4.6
Weight, kg	50.7 ± 7	50.1 ± 6.5	52.2 ± 6.3	49.5 ± 6.9	53.7 ± 7.6	50.2 ± 9.6	53.9 ± 8.7	50.8 ± 8.4
BMI, kg/m^2^	23 ± 3	24 ± 2	24 ± 3	23 ± 3	24 ± 3	23 ± 4	24 ± 3	24 ± 3.7
Parity, %								
Primiparous	30%	66.67%	54.55%	33.33%	50%	42.86%	37.5%	55.56%
Multiparous	70%	33.33%	45.55%	66.67%	50%	57.14%	62.5%	44.44%
Breastfeeding practices, %								
Exclusive	50%	55.56%	36.36%	61.11%	55%	40%	68.75%	31.58%
Predominant	50%	44.44%	63.64%	38.89%	15%	26.67%	12.5%	26.32%
Mixed	–	–	–	–	30%	20%	18.75%	31.58%
Subclinical mastitis Na : K^b^	0.43 ± 0.07	0.36 ± 0.5^*^	0.42 ± 0.07	0.40 ± 0.07	0.38 ± 0.91	0.39 ± 0.08	0.39 ± 0.11	0.38 ± 0.07
**Infant characteristics**								
Infant age, days	19 ± 8	28 ± 13^*^	24 ± 10	21 ± 10	147 ± 19	139 ± 21	141 ± 18	145 ± 21
Infant sex, %								
Male	55%	66.7%	63.6%	55.6%	35%	60%	25%	63.2%^*^
Female	45%	33.3%	36.4%	44.4%	65%	40%	75%	36.8%
Weight-for-age *Z*-score (WAZ)	−0.35 ± 0.57	−1.56 ± 0.53^***^	−0.39 ± 0.91	−0.93 ± 0.65	−0.23 ± 0.55	−1.66 ± 0.77^***^	−0.2 ± 0.64	−1.38 ± 0.86^***^
Length-for-age *Z*-score (LAZ)	−1.57 ± 1	−1.95 ± 0.74	−0.76 ± 0.61	−2.25 ± 0.56^***^	−1.21 ± 0.99	−2.82 ± 1.2^***^	−0.82 ± 0.73	−2.81 ± 1.02^***^
Head circumference-for-age *Z*-score (HCAZ)	−0.38 ± 0.9	−1.22 ± 1.27	−0.59 ± 1.39	−0.67 ± 0.88	−0.51 ± 1.01	−1.85 ± 1.18^*^	−0.49 ± 1.06	−1.65 ± 1.21^**^

a Comparisons were made between WAZ and LAZ subgroups at each stage of lactation: Wilcoxon rank-sum test; Wilcoxon rank-sum exact test; Fisher’s exact test; Pearson’s chi-squared test. ^*^p-value < 0.05, ^**^p-value < 0.001, ^***^p-value < 0.0001. There were missing values in the breastfeeding practices during late lactation: WAZ < -1SD: n=2 and LAZ< -1 SD: n=2.

b Subclinical mastitis is an asymptomatic inflammatory condition of the lactating breast diagnosed by the elevated sodium/potassium ratio (Na:K) >0.6 in milk.

### Human milk microbiome community

**Figure 1:**
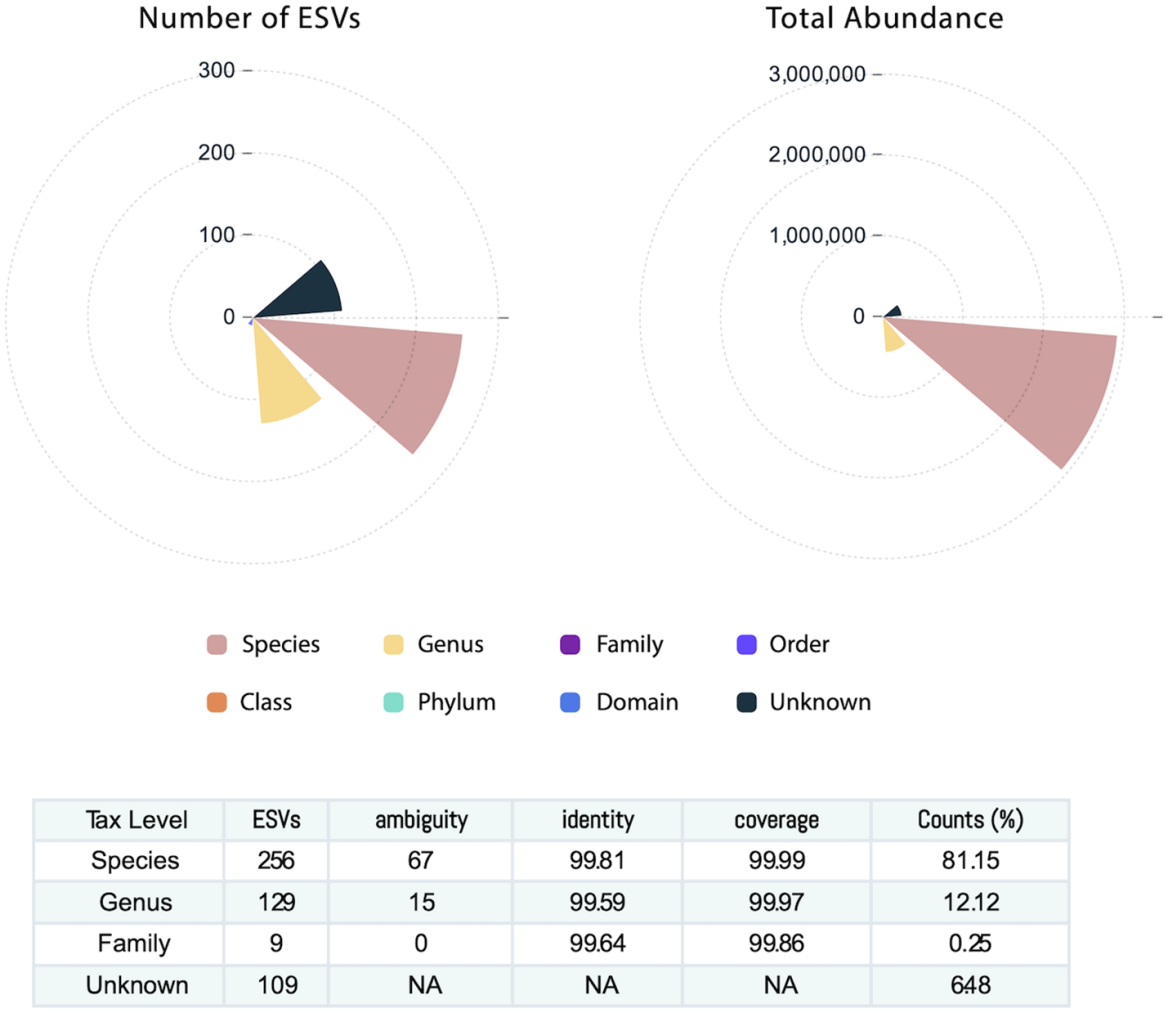
Human milk microbiome community. The number of exact sequence variants (ESVs) at different taxonomy levels showing that sequences annotated at the species level account for 81% of the ESVs. The total abundance is described at each taxonomy level.

Across the 64 human milk samples, 503 ESVs were assembled and captured 3,551,788 sequence reads. The identified 503 ESVs annotated 256 species accounting for 81.2% of reads, 129 genera, and 9 family or higher taxa in addition to 109 which could not be identified at >99% similarity in both identity and coverage to any known taxa and were classified as Unknowns (**Figure 1**). These Unknown taxa generally presented low abundance and contributed to only 6.5% of the total ESVs (**Figure 1**). The 256 ESVs annotated as putative species had an average BLASTn return for identity at 99.8% and for coverage at 99.9%.

**Figure 2:**
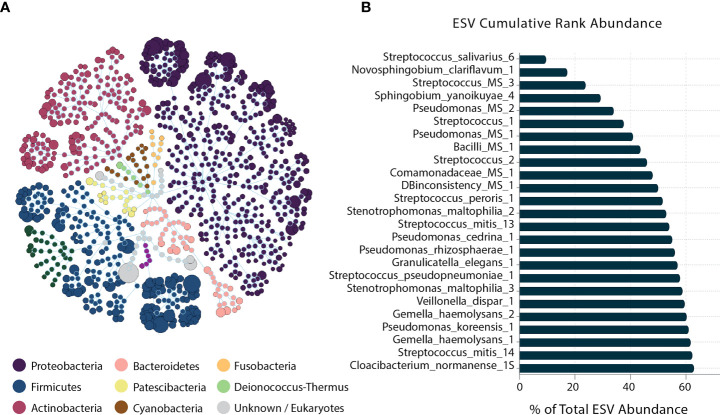
Human milk microbiome community overview. **(A)** The total microbial community colored at the phylum level. **(B)** Cumulative abundance of the 25 most abundant ESVs across samples.

At the phyla level, Proteobacteria, Firmicutes, and Actinobacteria presented the most prevalent phyla. At the species level, the ESV cumulative abundance revealed that 25 taxa of the 256 ESV annotated as putative species constituted 62.8% of the sequenced amplicons (**Figure 2A**). *Streptococcus_salivarius_6*, *Novosphingobium_clariflavum_1*, and *Streptococcus_MS_3* were the most abundant taxa across all samples, respectively.

Five of the 25 dominant taxa were DA in the different infant growth parameter groups: *Pseudomonas_MS_2*, *Streptococcus_mitis_13*, *Streptococcus_mitis_14*, *Pseudomonas_cedrina_1*, and *Stenotrophomonas_maltophilia_2. Pseudomonas_MS_2* was DA only in the normal weight (WAZ ≥ –1 SD) groups in both stages of lactation, and the non-stunted (LAZ ≥ –1.5 SD) group in late lactation. *Streptococcus_mitis_13* and *Streptococcus_mitis_14* were DA in the normal weight (WAZ ≥ –1 SD) group in late lactation. *Pseudomonas_cedrina_1* was DA in the normal groups: in the non-stunted (LAZ ≥ –1.5 SD) group in early lactation and in the normal weight group (WAZ ≥ –1 SD) in late lactation. Lastly, *Stenotrophomonas_maltophilia_2* was DA in the normal weight (WAZ ≥ –1 SD), whereas it was DA in the mildly stunted (LAZ < –1.5 SD) in early lactation (**Figure 2B**).

**Figure 3:**
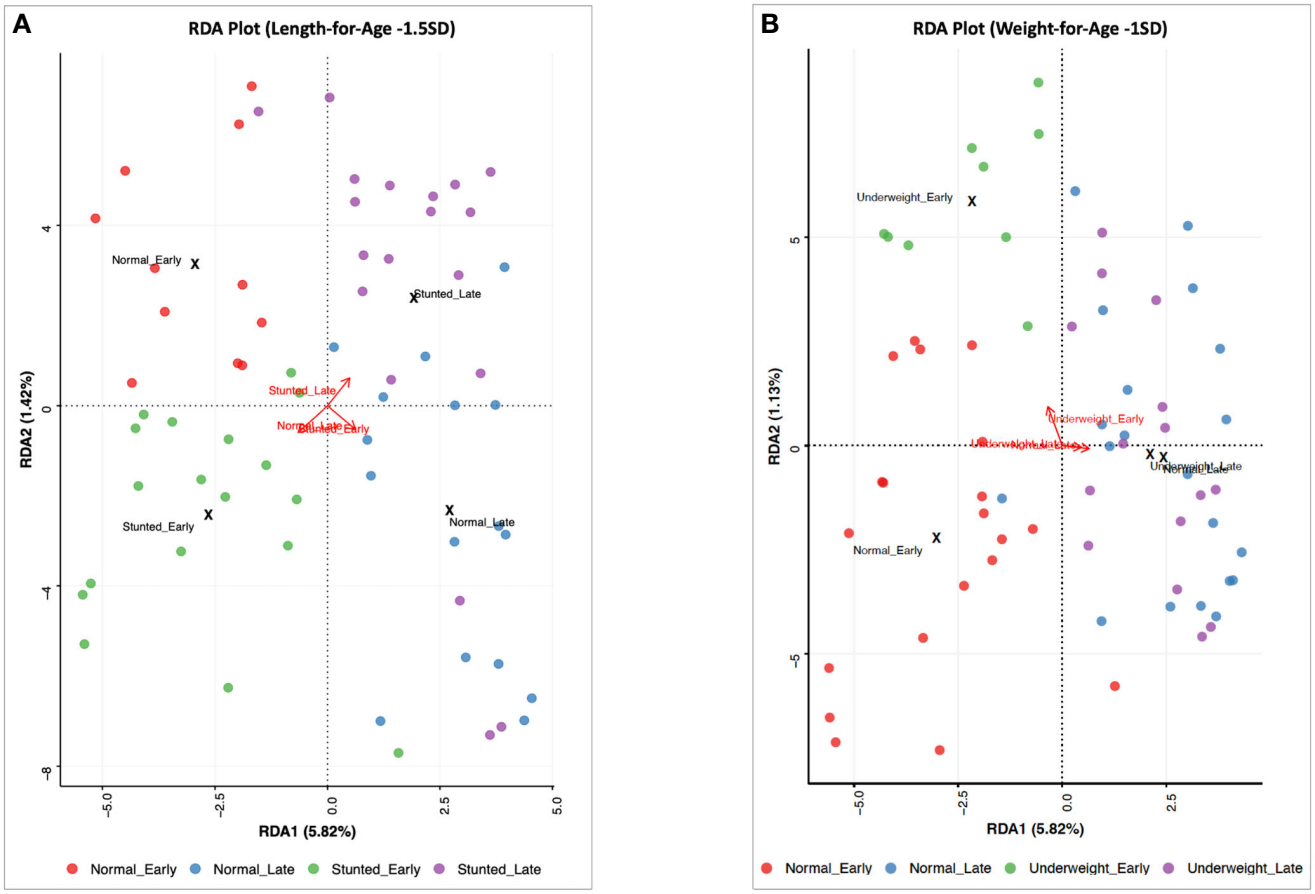
Redundancy analysis (RDA) and ordination representation of milk microbial community variation for **(A)** the non-stunted group [length-for-age *Z*-score (LAZ) ≥ −1.5SD) and the mildly stunted group (LAZ < −1.5SD) and **(B)** the normal weight group [weight-for-age *Z*-score (WAZ) ≥ −1SD] and the mildly underweight group (WAZ < −1SD) at the early and late stages of lactation (*p *= 0.004 and *p* < 0.001, respectively). RDA biplot showing individual samples colored by groups and vectors (in red) corresponding to the variable loadings (i.e., variable contribution). The black crosses represent the geometric center of each group. Colored circles show individual samples.

**Figure 4:**
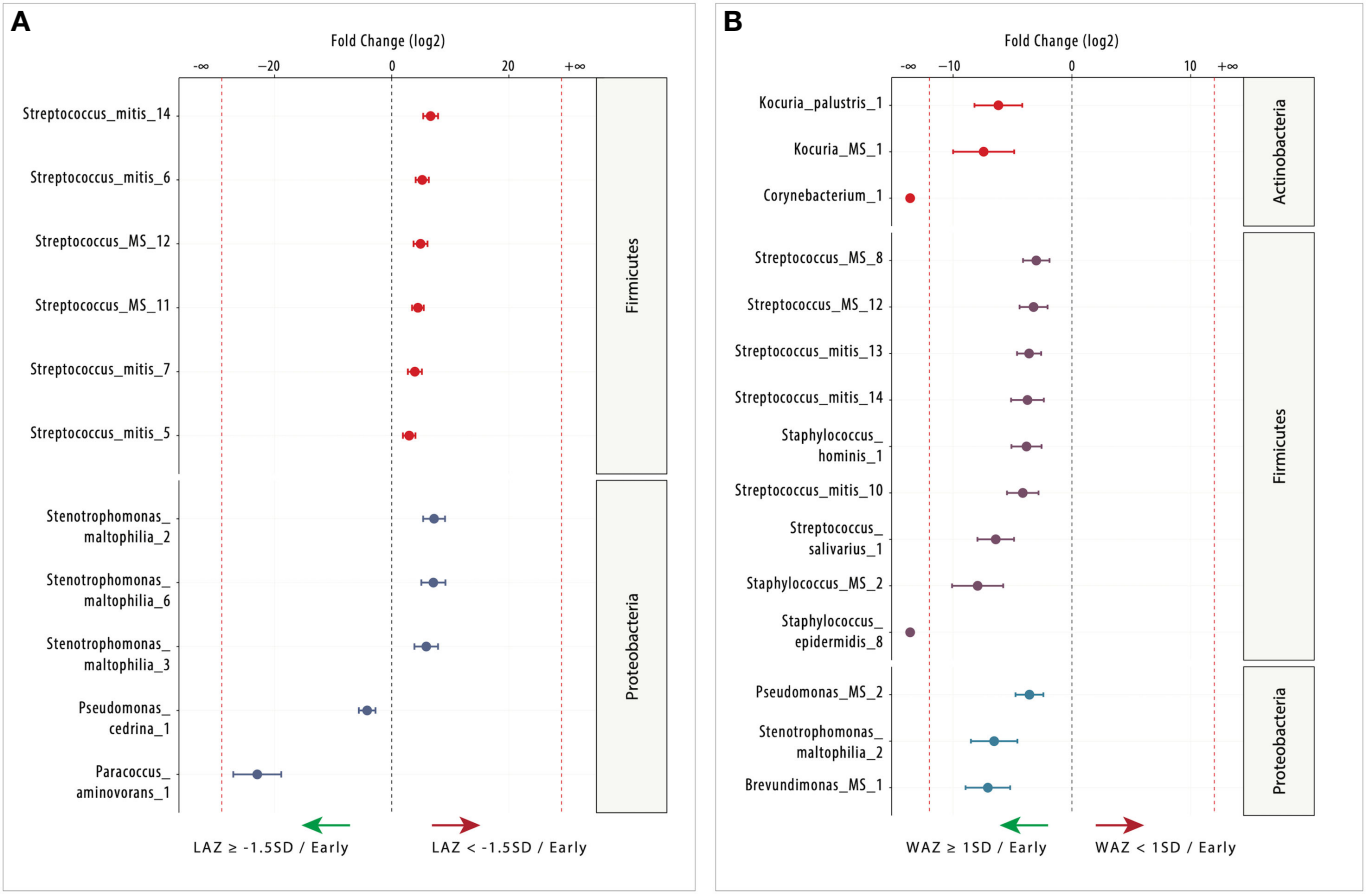
Differentially abundant (DA) ESVs associated with length-for-age *Z*-score (LAZ) and weight-for-age *Z*-score (WAZ) in early lactation. **(A)** Nine ESVs were significantly more abundant in the mildly stunted group (LAZ < −1.5SD: *n* = 18; right side) compared with two ESVs in the non-stunted group (LAZ ≥ -1.5SD *n* = 11; left side), and **(B)** 15 ESVs were significantly more abundant in the normal weight group (WAZ ≥ −1SD: *n* = 20; left side) compared with 0 ESV in the mildly underweight group (WAZ < −1SD: *n* = 9; right side). Species are colored and grouped by phylum. The dashed red line represents a limit beyond which ESVs were only quantified in a single group.

A comparison of human milk microbiota among infant WAZ and LAZ subgroups revealed some similarities and differences among DA taxa in these subgroups. On the one hand, in late lactation, four DA taxa occurred only in the normal groups (WAZ ≥ –1 SD and LAZ ≥ –1.5 SD): *Lactococcus_lactis_2*, *Streptococcus_MS_16*, *Streptococcus_salivarius_5*, and *Pseudomonas_MS_2*. On the other hand, five DA taxa differed between the normal weight (WAZ ≥ –1 SD) and the mildly stunted (LAZ < -1.5 SD) groups at both stages of lactation. *Stenotrophomonas_maltophilia_2*, *Streptococcus_mitis_14*, *Brevundimonas_MS_1*, *Streptococcus_mitis_10*, and *Streptococcus_MS_12* were DA taxa in the normal weight WAZ ≥ –1 SD group, but not in the mildly underweight (WAZ < –1 SD) group in early lactation. These same taxa were DA in the mildly stunted (LAZ < -1.5 SD) group; *Stenotrophomonas_maltophilia_2*, *Streptococcus_mitis_14*, and *Streptococcus_MS_12* were DA in the mildly stunted group (LAZ < –1.5 SD) in early lactation, whereas *Brevundimonas_MS_1* and *Streptococcus_mitis_10* were DA in the mildly stunted (LAZ < –1.5 SD) in late lactation. These taxa were not DA in the non-stunted (LAZ ≥ –1.5 SD) group (**Figures 3**, **4**).

#### Microbial diversity

The milk microbiome communities within each sample (i.e., alpha diversity) did not identify significant differences between the non-stunted (LAZ ≥ –1.5 SD) and the mildly stunted (LAZ < –1.5 SD) groups or between the normal weight (WAZ ≥ –1 SD) and mildly underweight (WAZ < –1 SD) groups either at early or at a late stage of lactation.

In contrast, beta diversity, through RDA, showed significant differences between the non-stunted (LAZ ≥ –1.5 SD) and the mildly stunted (LAZ < –1.5 SD) groups (*p* < 0.001) (**Figure 3A**) and between the normal weight (WAZ ≥ –1 SD) and the mildly underweight (WAZ < –1 SD) groups (*p* = 0.004) for both stages of lactation (**Figure 3B**).

### Differential abundance in early lactation

#### Infant length-for-age *Z*-score

In early lactation, 11 milk microbiome taxa were DA (FDR < 0.05) between non-stunted (LAZ ≥ –1.5 SD) and mildly stunted (LAZ < −1.5SD) (**Figure 4A**). The mildly stunted (LAZ < –1.5 SD) had more DA taxa accounting for nine DA taxa, mostly *Streptococcus* species, compared with only two DA taxa in the non-stunted (LAZ ≥ –1.5 SD). These taxa were annotated as species (nine ESVs) and genera (two ESVs). Most of these DA taxa in the mildly stunted belonged to the genus *Firmicutes* and were *Streptococcus* species including *Streptococcus_mitis_14* [fold change (FC) = 6.63], *Streptococcus_mitis_*6 (FC = 5.2), *Streptococcus_MS_12* (FC = 4.91), *Streptococcus_MS_11* (FC = 4.46), *Streptococcus_mitis_*7 (FC = 3.94), and *Streptococcus_mitis_5* (FC = 2.97). The other three DA species belonged to the *Proteobacteria* genus; they were *Stenotrophomonas_maltophilia_2* (FC = 7.22), *Stenotrophomonas_maltophilia_6* (FC = 7.1), and *Stenotrophomonas_maltophilia_3* (FC = 5.88). Only two DA species from the *Proteobacteria* genus were identified in the normal LAZ at early lactation; these included *Paracoccus_aminovorans_1* (FC = 22.94) and *Pseudomonas_cedrina_1* (FC = 4.19) (**Figure 4A**). None of the DA taxa in both groups belonged to the *Actinobacteria* genus in early lactation.

#### Infant weight-for-age *Z*-score

During early lactation, differential abundance analysis using DESeq2 identified 15 milk microbiome species that were DA (FDR < 0.05) between normal weight (WAZ ≥ –1 SD) and the mildly underweight (WAZ < –1 SD) (**Figure 4B**). All the 15 species were DA in the normal weight (WAZ ≥ –1 SD) group. The *Firmicutes* genera had nine DA species, namely, *Streptococcus_MS_8* (FC = 2.99), *Streptococcus_MS_12* (FC = 3.22), *Streptococcus_mitis_13* (FC = 3.60), *Streptococcus_mitis_14* (FC = 3.74), *Staphylococcus_hominis_1* (FC = 3.82), *Streptococcus_mitis_10* (FC = 4.13), *Streptococcus_salivarius_1* (FC = 6.41), *Staphylococcus_MS_2* (FC = 7.93), and *Staphylococcus_epidermidis_8* (FC = 23.67). The other species belonged to the *Actinobacteria* and *Proteobacteria* genera. Each genus had three DA species. *Kocuria_palustris_1* (FC = 6.18), *Kocuria_MS_1* (FC = 7.42), and *Corynebacterium_1* (FC = 22.39) belonged to the *Actinobacteria* genus, and *Brevundimonas_MS_1* (FC = 7.07), *Pseudomonas_MS_2* (FC = 3.57), and *Stenotrophomonas_maltophilia_2* (FC = 6.54) belonged to the *Proteobacteria* genus. There were two DA species that were only present in the normal weight group: *Corynebacterium_1* and *Staphylococcus_epidermidis_8* (**Figure 4B**).

### Differential abundance in late lactation

#### Infant length-for-age *Z*-score

**Figure 5:**
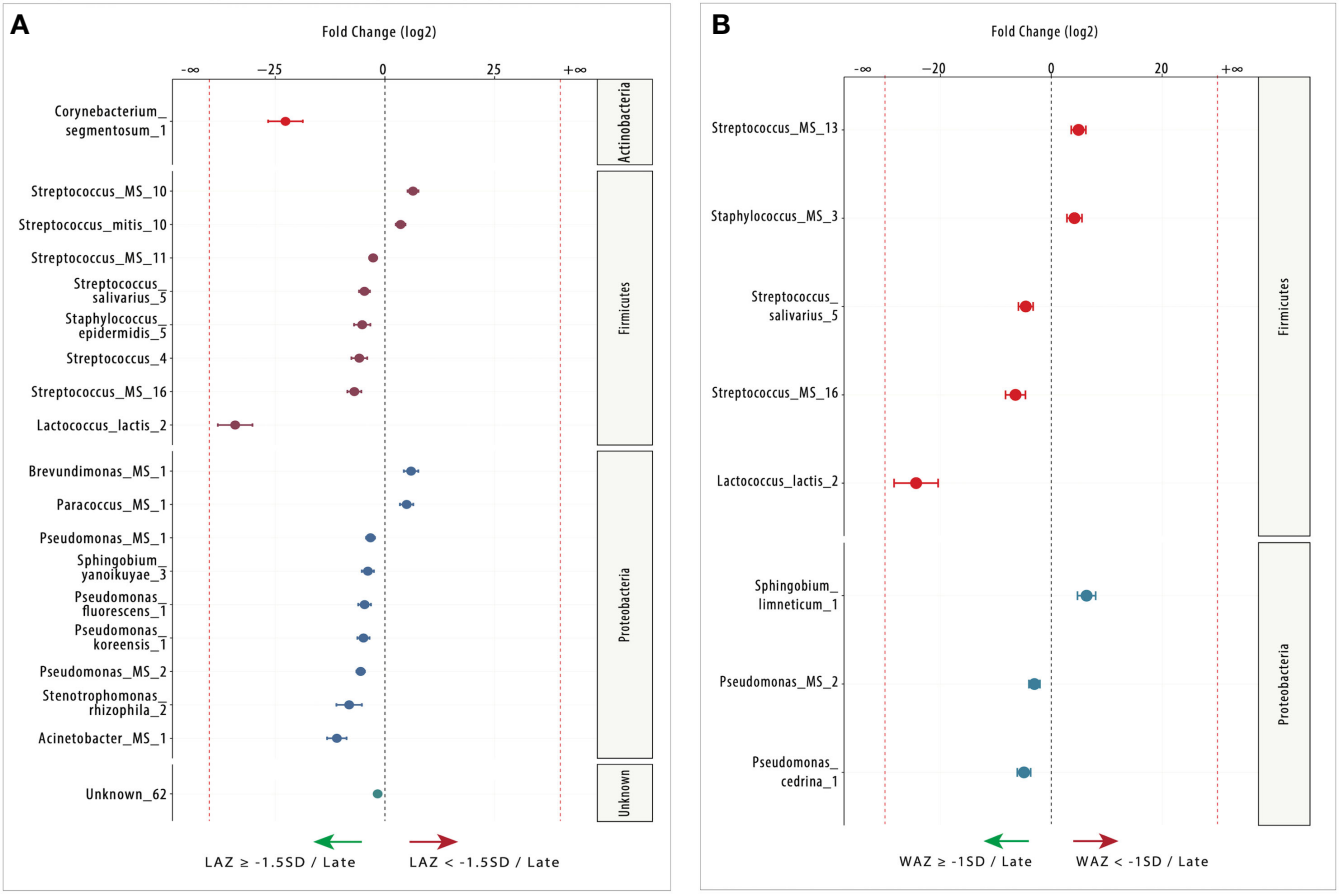
Differentially abundant (DA) ESVs associated with length-for-age *Z*-score (LAZ) and weight-for-age *Z*-score (WAZ) in late lactation. **(A)** Fifteen ESVs were significantly more abundant in the non-stunted group (LAZ ≥ −1.5SD: *n* = 16; left side) compared with four ESVs in the mildly stunted group (LAZ < - 1.5SD: *n* = 19; right side), and **(B)** five ESVs were significantly more abundant in the normal weight group (WAZ ≥ −1SD: *n* = 20; left side) compared with three ESVs in the mildly underweight group (WAZ < −1SD: *n* = 15; right side). Species are colored and grouped by phylum. The dashed red line represents a limit beyond which ESVs were only quantified in a single group.

In late lactation, there was a shift to more DA species in the non-stunted (LAZ ≥ –1.5 SD) group (**Figure 5A**). Out of the 19 DA taxa identified between the normal and mildly stunted groups, 14 were identified in the non-stunted (LAZ ≥ –1.5 SD) group. Most DA taxa belonged to the *Proteobacteria* genus including *Acinetobacter_MS_1* (FC = 10.95), *Stenotrophomonas_rhizophila_2* (FC = 8.14), *Pseudomonas_MS_2* (FC = 5.53), *Pseudomonas_koreensis_1* (FC = 4.88), *Pseudomonas_fluorescens_1* (FC = 4.63), *Sphingobium_yanoikuyae_3* (FC = 3.88), and *Pseudomonas_MS_1* (FC = 3.28), which was followed by the *Firmicutes* genus with *Lactococcus_lactis_2* (FC = 34.11), *Streptococcus_MS_16* (FC = 6.96), *Streptococcus_4* (FC = 5.84), *Staphylococcus_epidermidis_5* (FC = 5.17), *Streptococcus_salivarius_5* (FC = 4.66), and *Streptococcus_MS_11* (FC = 2.69). The normal group (LAZ ≥ –1.5 SD) had one Unknown DA taxa: Unknown_62 (FC = 1.66). The mildly stunted (LAZ < –1.5 SD) group had four DA species. Two species belonged to the Firmicutes genus, *Streptococcus_MS_10* (FC = 6.39) and *Streptococcus_mitis_10* (FC = 3.57), and two belonged to the Proteobacteria genus, *Brevundimonas_MS_1* (FC = 5.95) and *Paracoccus_MS_1* (FC = 4.94) (**Figure 5A**).

#### Infant weight-for-age *Z*-score

In late lactation, DESeq2 identified eight milk microbiome species that were DA (FDR < 0.05) between normal weight (WAZ ≥ –1 SD) and the mildly underweight (WAZ < –1 SD) groups. Five of the eight DA species were DA in the normal weight (WAZ ≥ –1 SD) group (**Figure 5B**). These species included three species that belonged to the *Firmicutes* genus, namely, *Streptococcus_salivarius_5* (FC = 4.60), *Streptococcus_MS_16* (FC = 6.45), and *Lactococcus_lactis_2* (FC = 24.38), and two species belonged to the *Proteobacteria* genus, namely, *Pseudomonas_MS_2* (FC = 3.03) and *Pseudomonas_cedrina_1* (FC = 4.92). The other three species were DA in the mildly underweight (WAZ < –1 SD) group; they included *Staphylococcus_MS_3* (FC = 4.17) and *Streptococcus_MS_13* (FC = 4.92), which belonged to the *Firmicutes* genus, and *Sphingobium_limneticum_1* (FC = 6.36), which belonged to the *Proteobacteria* genus. None of the DA species between the normal weight and the mildly underweight groups belonged to the *Actinobacteria* genus (**Figure 5B**).

## Discussion

Our findings identified several important relationships of the HMM with infant growth parameters. In general, there were shifts in the milk microbiota from early to late lactation as previously described (Gonzalez et al., 2021), but in this study, we uncovered further distinctions based on infant growth patterns. Our comparison of the HMM by infant weight (WAZ < –1 SD vs. WAZ ≥ –1 SD) and length (LAZ < –1.5 SD vs. LAZ ≥ –1.5 SD) in both early and late lactation identified four distinct human milk microbial clusters associated with infant growth before 6 months of age. First, in early lactation, all DA taxa were associated with infants having a WAZ ≥ –1 SD; none were identified as DA if infants were mildly underweight. Among these 15 DA species, six were *Streptococci*, several of which were ambiguous species, and three were *Staphylococci*, which are most often associated with the skin or the normal oropharyngeal cavity. Second, in late lactation, more DA species in milk were associated with WAZ ≥ –1 SD compared with WAZ < –1 SD. Both groups had normal flora, but the HMM of mothers of infants with WAZ ≥ –1 SD had more *Lactobacillales* species, whereas the HMM of mildly underweight infants (WAZ < –1 SD) in late lactation included more opportunistic bacteria. Third, with regard to LAZ in early lactation, the HMM of mildly stunted (LAZ < –1.5 SD) compared with non-stunted infants (LAZ ≥ –1 SD) had more DA taxa. Four of the nine DA species included four distinct *Streptococcus_mitis* ESVs, and three of the nine DA species included the environmental *Stenotrophomonas_maltophilia*; both *Streptococcus_mitis* and *Stenotrophomonas_maltophilia* are considered opportunistic bacteria. Fourth, in late lactation, the HMM of infants with LAZ ≥ –1.5 SD had 14 DA taxa, half of which were human colonizers including the LAB *Lactococcus_lactis_2*, compared with only 4 DA bacteria in the mildly stunted (LAZ < –1.5 SD), which were mainly ambiguous taxa that included both human colonizers and environmental taxa belonging to the Proteobacteria phylum (*Brevundimonas_MS_1* and *Paracoccus_MS_1*) associated with soil and water.

### Environmental bacteria in human milk

The presence of environmental bacteria in human milk is very common (Togo et al., 2019), especially where there is human interaction with the environment in rural agricultural and hunter-gatherer communities (Blum et al., 2019), which was the case for our study population. The latest systematic review reported that among 434 species in human milk, 277 (34%) were first isolated in the environment, 104 (13%) were associated with animals, and 53 (6%) were associated with plants (Togo et al., 2019). In our study, all infant groups with DA species at both stages of lactation had some DA taxa of environmental origin. Our analyses also identified several ambiguous environmental bacteria that were isolated from soil and water. Soil bacteria have shown beneficial growth-promoting functions that included antagonizing deleterious and pathogenic microorganisms, reducing inflammation (Schmidt et al., 2012; Silambarasan et al., 2020) and increasing microbial diversity in plants (Tasnim et al., 2017). In our study population, environmental bacteria were found in early lactation in both LAZ ≥ –1.5 SD and WAZ ≥ –1 SD and in both LAZ and WAZ subgroup categories in late lactation. The presence of environmental species in our Guatemalan mothers’ milk is supported by mothers’ participation in fruit and vegetable harvesting (Chomat et al., 2015) and their interaction with the Guatemalan-rich soil (Matsumoto et al., 2013) and can be considered as integral components of breast milk in agricultural societies.

### Associations of differentially abundant taxa with length-for-age *Z*-score

#### Early lactation LAZ

Few differentially abundant species were identified in the mother’s milk of infants with LAZ ≥ –1.5 SD in early lactation. Two, both of environmental origin, were observed. *Paracoccus_aminovorans_1* is a soil bacterium (Urakami et al., 1990; Czarnecki et al., 2017) and *Pseudomonas_cedrina_1* was previously isolated from grasses (Behrendt et al., 2009) and spring water (Dabboussi et al., 1998; Dabboussi et al., 1999). To date, the role of these bacteria in infant health has not been described. On the other hand, three of the nine DA species in the mildly stunted (LAZ < –1.5 SD) group had been previously characterized as opportunistic environmental *Stenotrophomonas_maltophilia* species (*Stenotrophomonas_maltophilia_2*, Stenotrophomonas*_maltophilia_3*, and *Stenotrophomonas_maltophilia_6*). *Stenotrophomonas_maltophilia* has been found on the skin and in the respiratory tract (Brooke, 2012; Gröschel et al., 2020), and we previously reported its presence in our human milk samples (Gonzalez et al., 2021) Moreover, it has been classified as an opportunistic bacteria (Brooke, 2012; Berg and Martinez, 2015) in children (Fedler et al., 2006; Brooke, 2012). Our findings provide evidence that specific HMM may be an overlooked source that can impact infant growth during early lactation.

The mildly stunted (LAZ < –1.5 SD) group also had four DA distinct oropharyngeal bacterial species associated with *Streptococcus_mitis.* These are the microorganisms responsible for the development of dental caries (Chava et al., 2012; Chaemsaithong et al., 2022). The four distinct *Streptococcus_mitis* ESVs found in mother’s milk were as follows: Streptococcus*_mitis_14*, *Streptococcus_mitis_5*, *Streptococcus_mitis_6*, and *Streptococcus_mitis_*7. *Streptococcus_mitis* has been identified as oropharynx bacteria (Kilian et al., 2014) and is also found in the infant oral microbiome within a few days after birth (Pearce et al., 1995). It is considered an opportunistic pathogen (Mitchell, 2011). 

There is evidence of emerging associations of pathogenic oral bacterial species with poor perinatal outcomes (Dasanayake et al., 2005; Siqueira et al., 2007; Srinivas et al., 2009; Shaw et al., 2016; Ren and Du, 2017; Robertson et al., 2019). Most women in our study population had periodontal disease, which was diagnosed through the detection of dental cavities (caries) and/or inflammation of the gum (gingivitis) (Chomat et al., 2015). Given that the maternal and infant oral ecosystems are important sources of human milk microbiota (Ramsay et al., 2004; Fernández et al., 2013), the dominance of the dental caries *Streptococcus_mitis* in the milk of mothers with the mildly stunted (LAZ < –1.5 SD) infants might suggest an association of poor oral health with unfavorable growth outcomes in early lactation. Thus, our data support an overlooked association between oral microbial dysbiosis with infant growth parameters in early lactation.

Finally, LAZ < –1.5 SD was also associated with two DA ambiguous taxa: *Streptococcus_MS_11* and *Streptococcus_MS_12*. Both ambiguous species were either the oropharyngeal bacterium *Streptococcus_mitis* or the emerging pathogenic bacterium *Streptococcus_pseudopneumoniae*, an overlooked pathogenic causative agent found in the lower respiratory tract, which highlights the presence of lower-respiratory tract infections in the community having a potential impact on early infant growth.

#### Late lactation LAZ

Several DA taxa identified in the non-stunted (LAZ ≥ –1.5 SD) group in late lactation were human colonizers including the LAB *Lactococcus_lactis_2*. Other human colonizers included firstly *Staphylococcus_epidermidis_5*, which is a ubiquitous normal human skin and mucous membrane colonizer that plays a role in balancing the epithelial microflora through maintaining a commensal relationship with the host through mechanical resistance (biofilm) and osmoprotection (Otto, 2009) and is described as a common human milk inhabitant (Heikkilä and Saris, 2003; Martín et al., 2007). Second, *Streptococcus_salivarius_5* is described as a normal infant oral microbiota (Pearce et al., 1995). As a human milk flora (Delorme et al., 2015), it has demonstrated inhibitory activities against *Staphylococcus* in human milk (Heikkilä and Saris, 2003). Third, *Corynebacterium_segmentosum_1* is a human gastric microbiota. *Corynebacterium* species have been detected in various habitats, including vegetables, dairy products, and soil (Brennan et al., 2001; Fudou et al., 2002). Most importantly, *Lactococcus_lactis_2*, which is known to inhibit the growth of *Staphylococcus* pathogens (Heikkilä and Saris, 2003; Gao et al., 2019), produces the bacteriocin nisin (Beasley and Saris, 2004), aids in lactose digestion (Klijn et al., 1995; Beasley and Saris, 2004), and can play a role in the bacteriological homeostasis of human milk (Heikkilä and Saris, 2003; Sharma et al., 2017).

Other DA taxa in the non-stunted (LAZ ≥ −1.5 SD) group were mainly ambiguous taxa of human colonizers and *Pseudomonas* species. One ambiguous taxon was *Acinetobacter_MS_1*, which included either the commensal human skin and mucosal colonizer *Acinetobacter_lwoffii* (formerly *Acinetobacter calcoaceticus* var. *lwoffii*) (Bouvet and Grimont, 1986; Ku et al., 2000), or the non-pathogenic environmental species *Acinetobacter_guillouiae*, which was obtained from soil and water (Yoon et al., 2014). Another ambiguous *Streptococci* human colonizer taxon was *Streptococcus_MS_11*, which can be either *Streptococcus_mitis* or *Streptococcus_pseudopneumoniae*, and *Streptococcus_MS_16*, which can be either *Streptococcus_mitis* or *Streptococcus_pneumoniae*, an oropharyngeal microflora (Pearce et al., 1995; Kilian et al., 2014) and a respiratory tract pathogen (Kilian et al., 2014), respectively. Although these species are causative agents of infections in human and pediatric patients (Mitchell, 2011; Kilian et al., 2014; Garriss et al., 2019), the presence of these potentially opportunistic species along with other pathogen-inhibitory species, such as *Lactococcus_lactis_2*, suggests a bacteriological homeostasis associated with the human milk ecosystem that is partially achieved through cross-talk between the different bacterial species of the human milk. This could include bacteriocin production to affect the growth of other species and/or the bacterial utilization of milk components, which might result in the protective effect of human milk and increased energy availability for breastfed infants.

The non-stunted (LAZ ≥ –1.5 SD) group also included DA *Stenotrophomonas* and *Pseudomonas* taxa of environmental origins. *Stenotrophomonas* species were *Stenotrophomonas_rhizophila_2* and *Sphingobium_yanoikuyae_3. Stenotrophomonas_rhizophila_2* has been associated with plant and rhizosphere of soils (Wolf et al., 2002). It is known for its plant growth-promoting features, mainly through antagonizing deleterious and pathogenic rhizosphere microorganisms (Schmidt et al., 2012; Pinski et al., 2020; Silambarasan et al., 2020). *Sphingobium_yanoikuyae_3* is a well-identified soil bioremediation species that possesses degradation activity in the environment (Cunliffe and Kertesz, 2006), but its role in human milk has not been investigated. *Pseudomonas* species were found associated with the HMM of infants with LAZ ≥ –1.5 SD in late lactation. They were *Pseudomonas_fluorescens_1*, *Pseudomonas_koreensis_1*, and the ambiguous taxa *Pseudomonas_MS_1* and *Pseudomonas_MS_2. Pseudomonas* are complex isolates of environmental sources and are frequently associated with soils and plants (Mauchline and Malone, 2017; Fluit et al., 2020). The fluorescent *Pseudomonas* represent a wide range of environmental species including soil rhizospheres and plant surfaces (Silby et al., 2009) that have been noted to promote plant health and to protect from pathogenic fungi (Haas and Défago, 2005). *Pseudomonas_fluorescens_1* was also identified in low abundance in the normal flora of various body sites, including the mouth, stomach, and lungs (Patel et al., 2013; Scales et al., 2014). *Pseudomonas fluorescens* also possesses functional traits that enable it to grow and thrive in mammalian hosts and that include the production of bioactive secondary metabolites (Gross and Loper, 2009). Second, *Pseudomonas_koreensis_1* is classified within the *P. fluorescens* complex (Gomila et al., 2015; Garrido-Sanz et al., 2016). *Pseudomonas_koreensis* was first isolated from agricultural soils (Lozano et al., 2017; Lopes et al., 2018) and rhizospheres (Lopes et al., 2018). It was also found in animal (yak) milk where it presented inhibitory antimicrobial activity and bioactivity against Gram-positive and Gram-negative bacteria (Kaur et al., 2019).

The other DA *Pseudomonas* were the ambiguous taxa *Pseudomonas_MS_1* and *Pseudomonas_MS_2*. Both taxa include mainly the environmental fluorescent *Pseudomonas* species of soil and water. *Pseudomonas_MS_1* is potentially one of the six species (*Pseudomonas_azotoformans*, *Pseudomonas_cedrina*, *Pseudomonas_fluorescens*, *Pseudomonas_libanensis*, *Pseudomonas_poae*, or *Pseudomonas_putida*). Some are oil and plant phyllosphere/rhizosphere species including *Pseudomonas_putida* (Volke et al., 2020), *Pseudomonas_azotoformans* (Heidari Nonakaran et al., 2015), *Pseudomonas_cedrina* (Behrendt et al., 2009), *Pseudomonas_fluorescens* (Silby et al., 2009), and *Pseudomonas_poae* (Behrendt et al., 2003). *Pseudomonas_poae* is capable of inhibiting the growth of *Penicillium expansum* and has demonstrated the capability to degrade its mycotoxin, patulin (Ren et al., 2021), which can cause digestive tract problems in human infants at high levels (Trucksess and Diaz-Amigo, 2011). Thus, the presence of *Pseudomonas_poae* in human milk might exert a protective effect in the breastfed infant’s gastrointestinal tract. *Pseudomonas_libanensis* has been isolated from spring water in Lebanon (Dabboussi et al., 1998; Dabboussi et al., 1999). The second ambiguous taxon was *Pseudomonas_MS_2*, which is either the *Pseudomonas_putida* or *Pseudomonas_gingeri*; these are soil species (Volke et al., 2020) and plant pathogenic species (Bahrami et al., 2018), respectively. Lastly, there was one Unknown ESV (Unknown_62) in the non-stunted (LAZ ≥ –1.5 SD) group at late lactation, which may emphasize the potential role of unidentified species in infant stunting.

In late lactation, only four taxa were DA in the mildly stunted (LAZ < –1.5 SD); these were mainly ambiguous taxa and included both human colonizers and environmental taxa. Of the two ambiguous taxa of environmental origin, two belonged to the Proteobacteria phylum (*Brevundimonas_MS_1* and *Paracoccus_MS_1*) that were associated with soil and water. *Brevundimonas_MS_1* was either *Brevundimonas_vesicularis* or *Brevundimonas_nasdae* that were isolated from the soil and aquatic environments, respectively (Rao and Kumar, 2014; Ryan and Pembroke, 2018; Stabler et al., 2018). The Proteobacteria phylum includes many potentially pathogenic species (Monira et al., 2011; Shin et al., 2015). However, the second DA taxon of the Proteobacteria phylum was an ambiguous environmental taxon; *Paracoccus_MS_1* can be one of two putative species: *Paracoccus_carotinifaciens* or *Paracoccus_marcusii.* Of interest to our findings is the recent evidence describing that *Paracoccus_marcusii* improved growth, elevated antioxidant properties, suppressed the expression of some inflammatory genes in marine animals (Yang et al., 2015; Xue et al., 2020), and increased the probiotic properties of whey proteins (Kalathinathan and Kodiveri Muthukaliannan, 2021).

Other human colonizers associated with LAZ < –1.5 SD were *Streptococcus_mitis_10* and *Streptococcus_MS_10*. *Streptococcus_mitis* is an oropharyngeal species (Pearce et al., 1995; Kilian et al., 2014) that has the potential to cause opportunistic infections (Mitchell, 2011). *Streptococcus_MS_10* can be one of the four species, namely, *Streptococcus_mitis*, *Streptococcus_oralis*, *Streptococcus_pneumoniae*, or *Streptococcus_pseudopneumoniae*, all of which cause respiratory infections in adult and pediatric patients (Kilian et al., 2014; Basaranoglu et al., 2019; Garriss et al., 2019).

### Associations of differentially abundant taxa associated with weight-for-age *Z*-score

#### Early lactation WAZ

Most of the DA in the human milk microbiome of infants with WAZ ≥ –1 SD were normal human microflora and human milk colonizers. Commensal bacteria found in milk were *Streptococcus mitis*, *Kocuria_palustris_1*, and *Streptococcus_salivarius_1. Streptococcus mitis* and *Kocuria_palustris_1* are common oropharyngeal species (Pearce et al., 1995; Kilian et al., 2014). *Kocuria_palustris_1* is a normal flora of the skin and mucous membranes of the mouth and digestive and genital tracts in humans and animals (Stackebrandt et al., 1995). Generally, *Kocuria* are commonly considered non-pathogenic and have been rarely associated with human infections (Kandi et al., 2016).

Additionally, there were two human milk colonizers: *Streptococcus_salivarius_1*, which has been isolated from human milk (Delorme et al., 2015) and from the oral cavity of full-term infants during early lactation (Pearce et al., 1995), and *Staphylococcus epidermidis*, which is a normal colonizer of human skin and mucous membranes (Otto, 2009). *Staphylococcus epidermidis* may provide a protective role in balancing epithelial microflora and serve as a reservoir of resistance genes (Otto, 2009). Generally, it is considered a healthy human milk microflora and, in particular, has been shown to inhibit *Staphylococcus aureus* in expressed human milk *in vitro* (Heikkilä and Saris, 2003; Martín et al., 2007). Although *S. epidermidis* can be involved in infections (Otto, 2009; Coates et al., 2014), it can be regulated and inhibited by other milk bacterial species (Delgado et al., 2009; Diepers et al., 2017). Moreover, during the first month of life, human milk *Staphylococcus_epidermidis* strains can outcompete more virulent strains in the infant’s gut and appear to be protective (Soeorg et al., 2013; Soeorg et al., 2017).

Several other DA taxa associated with WAZ ≥ –1 SD were ambiguous taxa with the possibility of being both a normal flora and/or opportunistic bacteria. Among these were *Streptococcus_MS_8*, *Streptococcus_MS_12*, and *Streptococcus_MS_2*. Two of them (*Streptococcus_MS_8* and *Streptococcus_MS_12*) were either one of two species: a commensal oropharyngeal inhabitant *Streptococcus_mitis* (Kilian et al., 2014) or *Streptococcus_pseudopneumoniae*, an emerging causative agent of lower-respiratory tract infections (Garriss et al., 2019). The third ambiguous taxon *Staphylococcus_MS_2* was one of three species (*Staphylococcus_epidermidi*, *Staphylococcus_haemolyticus*, or *Staphylococcus_hominis*). All are often described as human skin microflora (Kloos and Musselwhite, 1975; Heikkilä and Saris, 2003; Diepers et al., 2017). Although they have been associated with human nosocomial infections, these coagulase-negative *Staphylococci* tend to be less pathogenic (Bennett et al., 2019).

We also observed ambiguous environmental taxa that were associated with WAZ ≥ –1 SD: *Brevundimonas_MS_1*, *Pseudomonas_MS_2*, and *Kocuria_MS_1*. First, *Brevundimonas_MS_1* was either one of two aquatic environmental species: *Brevundimonas_nasdae* (Rao and Kumar, 2014) or *Brevundimonas_vesicularis*, which are rarely known to cause infections in humans unless in immunocompromised hosts (Ryan and Pembroke, 2018; Stabler et al., 2018). Second, *Pseudomonas_MS_2* was either the soil species *Pseudomonas_putida* (Volke et al., 2020) or the plant pathogenic species *Pseudomonas_gingeri* (Bahrami et al., 2018). Third, *Kocuria_MS_1* was either *Kocuria_indica* or *Kocuria_marina*. Both species have been isolated from sediment and marine sediment samples (Kim et al., 2004; Dastager et al., 2014), respectively, and generally are considered non-pathogenic (Kandi et al., 2016).

The last DA taxon in the normal weight group (WAZ ≥ –1 SD) was the environmental pathogenic bacterium *Stenotrophomonas_maltophilia_2* (Brooke, 2012; Berg and Martinez, 2015), which is an environmental bacterium that has been isolated from water and soil (Adjidé et al., 2009) and from animals and plant materials (Berg et al., 2005; Furushita et al., 2005). It was previously isolated from raw milk and human feces (Spencer, 1995). Although it has been classified as a multidrug-resistant global opportunistic pathogen (Brooke, 2012), this pathogenicity is only associated with immunoincompetent individuals (Smeets et al., 2007; Adegoke et al., 2017), which was not the case in our study.

#### Late lactation WAZ

The normal weight (WAZ ≥ –1 SD) group included two normal human microflora species: *Streptococcus_salivarius_5* and *Lactococcus_lactis_2*. First, *Streptococcus_salivarius_5* is a commensal microflora that was isolated from the oral cavity of full-term infants during the first month (Pearce et al., 1995) and is commonly isolated from milk (Delorme et al., 2015). Second, *Lactococcus_lactis_2* belongs to the genus *Lactobacillus* and has been isolated from human milk (Beasley and Saris, 2004). The *Lactobacillus* genus is known to have multidirectional health-promoting effects for breastfed infants (Łubiech and Twarużek, 2020). RCTs with *Lactobacillus* supplementation of nursing mothers have reported increased infant WAZ but not LAZ (Pastor-Villaescusa et al., 2020) and greater weight and height gain in infants of supplemented mothers at 1 year of age (Mantaring et al., 2018). *Lactococcus_lactis* has demonstrated effective inhibition against *Staphylococcus aureus* (Heikkilä and Saris, 2003; Gao et al., 2019) and can produce nisin (Beasley and Saris, 2004). Nisin can inhibit bacterial growth (Naidu, 2000), and is now recognized as limiting the growth of bacterial pathogens (Soltani et al., 2020). Thus, it is possible that the bacteriocin nisin may play a role in the bacteriological homeostasis of human milk (Heikkilä and Saris, 2003; Sharma et al., 2017). Additionally, *Lactococcus_lactis* was able to metabolize lactose and different forms of carbohydrates (Beasley and Saris, 2004), suggesting a possible probiotic role in carbohydrate digestion in the infant’s gastrointestinal tract (Klijn et al., 1995; Hussain et al., 2021).

Other DA species in milk associated with growth in normal weight (WAZ ≥ –1 SD) infants were environmental and ambiguous taxa. These included the ambiguous taxa *Streptococcus_MS_16*, which was either *Streptococcus_mitis* or *Streptococcus_pneumoniae*, and *Pseudomonas_MS_2*, which was one of two environmental species (*Pseudomonas_putida* or *Pseudomonas_gingeri*) (Bahrami et al., 2018; Volke et al., 2020). The last DA species was the environmental bacterium *Pseudomonas_cedrina_1*, which has been isolated from grasses (Behrendt et al., 2009) and spring water (Dabboussi et al., 1998; Dabboussi et al., 1999).

The mildly underweight group (WAZ < –1 SD) had three DA taxa of different origins; however, one of them included an ambiguous taxon with potential oral pathogenicity. First, *Sphingobium_limneticum_1* is an environmental bacterium that was isolated from an aquatic environment (Chen et al., 2013). The other two were the ambiguous taxa *Staphylococcus_MS_3*, which was either the human skin microflora species *Staphylococcus_haemolyticus* or *Staphylococcus_hominis* (Kloos and Musselwhite, 1975; Heikkilä and Saris, 2003), or *Streptococcus_MS_13*, which was one of the three oral microbiome species with potential pathogenicity (*Streptococcus_timonensis*, *Streptococcus_oralis*, or *Streptococcus_cristatus*). *Streptococcus_timonensis* has recently been associated with the oropharynx mucosa of a healthy 5-year-old child (Qi et al., 2021). *Streptococcus_oralis* is a commensal bacteria of the normal human oral microbiota (Reichmann et al., 2011). It belongs to the *Mitis* group, which contains *Streptococcus_pneumoniae*, a major human pathogen that is capable of opportunistic pathogenicity (Do et al., 2009; Basaranoglu et al., 2019). *Streptococcus_cristatus* has been isolated from human throats and oral cavities including coronal dental plaque and periodontal abscess. Evidence supports the negative association of oral bacteria involved in dental plaque (Gutiérrez-Venegas et al., 2019) with infant birth weight (Dasanayake et al., 2005; Siqueira et al., 2007). Our findings suggest the potential impacts of these potential oral pathogenic bacteria in the human milk microbiome on infant weight during late lactation.

## Limitations and strengths

We recognize that our study had some limitations. In this study, we used the 27F/533R primer, which can amplify the core human milk genus *Cutibacterium* (Hunt et al., 2011; Jost et al., 2013; Jiménez et al., 2015) but cannot amplify species from the genus *Bifidobacterium* (Klindworth et al., 2013), which is not always identified in human milk studies using such primers (Davé et al., 2016; Ramani et al., 2018) as was the case in our study population. Sample size issues were examined from two perspectives. Originally, this cross-sectional study had been powered to detect only differences in infant growth in early and late lactation and not differences for stunting and underweight; thus, cut points for *Z*-scores were adapted to equalize sample sizes for microbial analyses. What we describe here are associative correlates with human growth. It is always important to accept a caveat of causal agnosticism. That is, we cannot say here whether the differential abundance patterns had any effects on promoting or retarding growth, or whether the differential size of the offspring influences the composition of the maternal HMM, or whether unmeasured factors impinging on the maternal–infant dyad gave rise to both HMM differences and variations in size.

Genome Quebec confirmed that sample extraction yielded sufficient DNA to capture more than 6 million sequence reads across all breast milk samples which allowed us to proceed with our secondary analyses (Gonzalez et al., 2021). Our methods were designed for improved species-level microbial identification with the criteria of >99% for identity and coverage (Gonzalez et al., 2019); thus, our analysis uncovered DA ambiguous species (*_MS*). However, with these adjustments, we were able to identify four distinct clusters of microbial communities using the modified anthropometric indices by stage of lactation. Other strengths include the observation that despite evidence of growth faltering in the first 6 months postpartum, all mothers complied with the WHO recommendations to breastfeed for 6 months.

## Conclusion

In conclusion, impaired infant growth in breastfed infants during the first 6 months of lactation was associated with greater DA of opportunistic and pathogenic bacterial species in human milk, whereas the microbiome of infants with either LAZ > –1.5 SD or WAZ > –1 SD had greater differential abundance of oral commensal and lactic acid bacteria. Moreover, bacterial species commonly found in the environment were present in mother’s milk and impacted infant growth. Finally, our findings highlight an overlooked contribution of the HMM to early infant growth and demonstrate an understudied role of the HMM with infant growth faltering in *Mam-*Mayan Indigenous communities in Guatemala during the first 6 months of lactation.

## Data availability statement

All data are made available within the manuscript or **Supplementary Data**. Raw sequence data has been deposited at the European Genome-Phenome Archive (EGAD00001004160) and are available upon request to KK, kristine.koski@mcgill.ca.

## Ethics statement

McGill Institutional Review Board and CeSSIAM Human Subjects Committee reviewed and approved the studies involving human participants. All participating mothers provided written informed consent for participation. Written informed consent to participate in this study was provided by the participants’ legal guardian/next of kin.

## Author contributions

TA drafted the manuscript. TA and EG performed the statistical analyses. EG analyzed the microbiome data and created the figures. NS provided funding and supervised the field data collection. KK provided funding for the 16S RNA analysis and, together with TA, framed the study design. All authors contributed to the article and approved the submitted version.

## Funding

This work was supported by the Natural Sciences and Engineering Research Council of Canada Discovery (Grant #RGPIN-2016-0496) (KK).

## Acknowledgments

Special thanks to McGill University and Genome Quebec for technical assistance with 16S rRNA sequencing. We thank H. Wren, AM Chomat, and the CeSSIAM field team for sample collection and the participating mothers for providing valuable milk samples.

## Conflict of interest

The authors declare that the research was conducted in the absence of any commercial or financial relationships that could be construed as a potential conflict of interest.

## Publisher’s note

All claims expressed in this article are solely those of the authors and do not necessarily represent those of their affiliated organizations, or those of the publisher, the editors and the reviewers. Any product that may be evaluated in this article, or claim that may be made by its manufacturer, is not guaranteed or endorsed by the publisher.

## Supplementary material

Supplementary File 1
*Decontam* run on this dataset identified one OUT (*Bradyrhizobium_MS_2*) as putative contamination out of 1505. This out was deselected by DESeq2 and was excluded as a candidate for differential abundance.

## Abbreviations

DA, differentially abundant; ESV, exact sequence variant; HC, head circumference; HCAZ, head circumference-for-age *Z*-score; HMM, human milk microbiome; HMO, human milk oligosaccharides; LAZ, length-for-age *Z*-score; WAZ, weight-for-age *Z*-score.

## References

[B1] Adegoke A. A. Stenström T. A. Okoh A. I. (2017). Stenotrophomonas maltophilia as an emerging ubiquitous pathogen: Looking beyond contemporary antibiotic therapy. Front. Microbiol. 8, 2276. doi: 10.3389/fmicb.2017.02276 29250041 PMC5714879

[B2] Adjidé C. De Meyer A. Weyer M. Obin O. Lamory F. Lesueur C.. (2009). Stenotrophomonas maltophilia and pseudomonas aeruginosa water-associated microbiologic risk assessment in amiens' university hospital centre. Pathologie-biologie 58 (2), e1–e5. doi: 10.1016/j.patbio.2009.07.006 19892487

[B3] Akcaboy M. Malbora B. Zorlu P. Altınel E. Oguz M. M. Senel S. (2015). Vitamin B12 deficiency in infants. Indian J. Pediatr. 82 (7), 619–624. doi: 10.1007/s12098-015-1725-3 25840526

[B4] Arrieta M.-C. Stiemsma L. T. Amenyogbe N. Brown E. M. Finlay B. (2014). The intestinal microbiome in early life: Health and disease. Front. Immunol. 5, 427. doi: 10.3389/fimmu.2014.00427 25250028 PMC4155789

[B5] Bahrami T. Zarvandi S. De Mot R. Gross H. Changi-Ashtiani M. Shahani T.. (2018). Draft genome sequence of pseudomonas gingeri strain LMG 5327, the causative agent of ginger blotch in agaricus bisporus. Genome announce. 6 (13), e00196–e00118. doi: 10.1128/genomeA.00196-18 PMC5876480 29599158

[B6] Bank U. W. W. (2020). Joint child malnutrition estimates, 2020 edition. Available at: https://data.unicef.org/resources/jme-report-2020/.

[B7] Basaranoglu S. T. Ozsurekci Y. Aykac K. Aycan A. E. Bıcakcigil A. Altun B.. (2019). Streptococcus mitis/oralis causing blood stream infections in pediatric patients. Jpn J. Infect. Dis. 72 (1), 1–6. doi: 10.7883/yoken.JJID.2018.074 30175731

[B8] Beasley S. S. Saris P. E. (2004). Nisin-producing lactococcus lactis strains isolated from human milk. Appl. Environ. Microbiol. 70 (8), 5051–5053. doi: 10.1128/AEM.70.8.5051-5053.2004 15294850 PMC492443

[B9] Behrendt U. Schumann P. Meyer J. M. Ulrich A. (2009). Pseudomonas cedrina subsp. fulgida subsp. nov., a fluorescent bacterium isolated from the phyllosphere of grasses; emended description of pseudomonas cedrina and description of pseudomonas cedrina subsp. cedrina subsp. nov. Int. J. Syst. Evol. Microbiol. 59 (Pt 6), 1331–1335. doi: 10.1099/ijs.0.005025-0 19502311

[B10] Behrendt U. Ulrich A. Schumann P. (2003). Fluorescent pseudomonads associated with the phyllosphere of grasses; pseudomonas trivialis sp. nov., pseudomonas poae sp. nov. and pseudomonas congelans sp. nov. Int. J. Syst. Evol. Microbiol. 53 (Pt 5), 1461–1469. doi: 10.1099/ijs.0.02567-0 13130034

[B11] Bennett J. E. Dolin R. Blaser M. J. (2019). Mandell, Douglas, and bennett's principles and practice of infectious diseases e-book (Philadelphia, PA: Elsevier/Saunders).

[B12] Berg G. Eberl L. Hartmann A. (2005). The rhizosphere as a reservoir for opportunistic human pathogenic bacteria. Environ. Microbiol. 7 (11), 1673–1685. doi: 10.1111/j.1462-2920.2005.00891.x 16232283

[B13] Berg G. Martinez J. L. (2015). Friends or foes: Can we make a distinction between beneficial and harmful strains of the stenotrophomonas maltophilia complex? Front. Microbiol. 6, 241. doi: 10.3389/fmicb.2015.00241 25873912 PMC4379930

[B14] Blum W. E. Zechmeister-Boltenstern S. Keiblinger K. M. (2019). Does soil contribute to the human gut microbiome? Microorganisms 7 (9), 287. doi: 10.3390/microorganisms7090287 31450753 PMC6780873

[B15] Bouvet P. J. Grimont P. A. (1986). Taxonomy of the genus acinetobacter with the recognition of acinetobacter baumannii sp. nov., acinetobacter haemolyticus sp. nov., acinetobacter johnsonii sp. nov., and acinetobacter junii sp. nov. and emended descriptions of acinetobacter calcoaceticus and acinetobacter lwoffii. Int. J. Syst. Evol. Microbiol. 36 (2), 228–240. doi: 0020-7713/86/020228-13$02.00/0

[B16] Brennan N. M. Brown R. Goodfellow M. Ward A. C. Beresford T. P. Simpson P. J.. (2001). Corynebacterium mooreparkense sp. nov. and corynebacterium casei sp. nov., isolated from the surface of a smear-ripened cheese. Int. J. Syst. Evol. Microbiol. 51 (3), 843–852. doi: 10.1099/00207713-51-3-843 11411705

[B17] Brooke J. S. (2012). Stenotrophomonas maltophilia: An emerging global opportunistic pathogen. Clin. Microbiol. Rev. 25 (1), 2–41. doi: 10.1128/CMR.00019-11 22232370 PMC3255966

[B18] Brown K. Henretty N. Chary A. Webb M. F. Wehr H. Moore J.. (2016). Mixed-methods study identifies key strategies for improving infant and young child feeding practices in a highly stunted rural indigenous population in G uatemala. Maternal Child Nutr. 12 (2), 262–277. doi: 10.1111/mcn.12141 PMC6860048 25040768

[B19] Cabrera-Rubio R. Collado M. C. Laitinen K. Salminen S. Isolauri E. Mira A. (2012). The human milk microbiome changes over lactation and is shaped by maternal weight and mode of delivery. Am. J. Clin. Nutr. 96 (3), 544–551. doi: 10.3945/ajcn.112.037382 22836031

[B20] Cargo M. Mercer S. L. (2008). The value and challenges of participatory research: Strengthening its practice. Annu. Rev. Public Health 29, 325–350. doi: 10.1146/annurev.publhealth.29.091307.083824 18173388

[B21] Carney M. C. Zhan X. Rangnekar A. Chroneos M. Z. Craig S. J. Makova K. D.. (2021). Associations between stool micro-transcriptome, gut microbiota, and infant growth. J. Dev. Orig. Health Dis. 12 (6), 876–882, 1–7. doi: 10.1017/S2040174420001324 33407969 PMC8675179

[B22] Chaemsaithong P. Lertrut W. Kamlungkuea T. Santanirand P. Singsaneh A. Jaovisidha A.. (2022). Maternal septicemia caused by streptococcus mitis: A possible link between intra-amniotic infection and periodontitis. case report and literature review. BMC Infect. Dis. 22 (1), 1–9. doi: 10.1186/s12879-022-07530-z 35725441 PMC9208128

[B23] Chava V. R. Manjunath S. Rajanikanth A. Sridevi N. (2012). The efficacy of neem extract on four microorganisms responsible for causing dental caries viz streptococcus mutans, streptococcus salivarius, streptococcus mitis and streptococcus sanguis: an *in vitro* study. J. Contemp. Dent. Pract. 13 (6), 769–772. doi: 10.5005/jp-journals-10024-1227 23404001

[B24] Chen H. Jogler M. Rohde M. Klenk H. P. Busse H. J. Tindall B. J.. (2013). Sphingobium limneticum sp. nov. and sphingobium boeckii sp. nov., two freshwater planktonic members of the family sphingomonadaceae, and reclassification of sphingomonas suberifaciens as sphingobium suberifaciens comb. nov. Int. J. Syst. Evol. Microbiol. 63 (Pt 2), 735–743. doi: 10.1099/ijs.0.040105-0 22561591

[B25] Chomat A. M. Solomons N. W. Koski K. G. Wren H. M. Vossenaar M. Scott M. E. (2015). Quantitative methodologies reveal a diversity of nutrition, infection/illness, and psychosocial stressors during pregnancy and lactation in rural mam-Mayan mother–infant dyads from the Western highlands of Guatemala. Food Nutr. Bull. 36 (4), 415–440. doi: 10.1177/0379572115610944 26481796

[B26] Coates R. Moran J. Horsburgh M. J. (2014). Staphylococci: Colonizers and pathogens of human skin. Future Microbiol. 9 (1), 75–91. doi: 10.2217/fmb.13.145 24328382

[B27] Colombara D. V. Hernández B. Gagnier M. C. Johanns C. Desai S. S. Haakenstad A.. (2015). Breastfeeding practices among poor women in mesoamerica. J. Nutr. 145 (8), 1958–1965. doi: 10.3945/jn.115.213736 26136592

[B28] Cunliffe M. Kertesz M. A. (2006). Effect of sphingobium yanoikuyae B1 inoculation on bacterial community dynamics and polycyclic aromatic hydrocarbon degradation in aged and freshly PAH-contaminated soils. Environ. pollut. 144 (1), 228–237. doi: 10.1016/j.envpol.2005.12.026 16524654

[B29] Czarnecki J. Dziewit L. Puzyna M. Prochwicz E. Tudek A. Wibberg D.. (2017). Lifestyle-determining extrachromosomal replicon pAMV1 and its contribution to the carbon metabolism of the methylotrophic bacterium paracoccus aminovorans JCM 7685. Environ. Microbiol. 19 (11), 4536–4550. doi: 10.1111/1462-2920.13901 28856785

[B30] Dabboussi F. Hamze M. Elomari M. Verhille S. Baida N. Izard D.. (1998). A numerical study of fluorescent pseudomonas strains isolated from three Lebanese spring waters. J. européen d’hydrol. 28 (3), 325–338. doi: 10.1051/water/19982803325

[B31] Dabboussi F. Hamze M. Elomari M. Verhille S. Baida N. Izard D.. (1999). Taxonomic study of bacteria isolated from Lebanese spring waters: Proposal for pseudomonas cedrella sp. nov. and p. orientalis sp. nov. Res. Microbiol. 150 (5), 303–316. doi: 10.1016/s0923-2508(99)80056-4 10422691

[B32] Daniels L. Gibson R. S. Diana A. Haszard J. J. Rahmannia S. Luftimas D. E.. (2019). Micronutrient intakes of lactating mothers and their association with breast milk concentrations and micronutrient adequacy of exclusively breastfed Indonesian infants. Am. J. Clin. Nutr. 110 (2), 391–400. doi: 10.1093/ajcn/nqz047 31152543 PMC6669051

[B33] Dasanayake A. P. Li Y. Wiener H. Ruby J. D. Lee M. J. (2005). Salivary actinomyces naeslundii genospecies 2 and lactobacillus casei levels predict pregnancy outcomes. J. periodontol. 76 (2), 171–177. doi: 10.1902/jop.2005.76.2.171 15974839

[B34] Dastager S. G. Tang S. K. Srinivasan K. Lee J. C. Li W. J. (2014). Kocuria indica sp. nov., isolated from a sediment sample. Int. J. Syst. Evol. Microbiol. 64 (Pt 3), 869–874. doi: 10.1099/ijs.0.052548-0 24254742

[B35] Davé V. Street K. Francis S. Bradman A. Riley L. Eskenazi B.. (2016). Bacterial microbiome of breast milk and child saliva from low-income Mexican-American women and children. Pediatr. Res. 79 (6), 846–854. doi: 10.1038/pr.2016.9 26756784 PMC4899194

[B36] Davis J. C. Lewis Z. T. Krishnan S. Bernstein R. M. Moore S. E. Prentice A. M.. (2017). Growth and morbidity of Gambian infants are influenced by maternal milk oligosaccharides and infant gut microbiota. Sci. Rep. 7 (1), 1–16. doi: 10.1038/srep40466 28079170 PMC5227965

[B37] Davis E. Musaad S. Monaco M. Donovan S. (2019). Early life nutrient intake is associated with weight-for-Length z-scores at 3 and 12 months (P11-127-19). Curr. Dev. Nutr. 3 (Supplement_1). doi: 10.1093/cdn/nzz048.P11-127-19

[B38] Delgado S. Arroyo R. Jiménez E. Marín M. L. del Campo R. Fernández L.. (2009). Staphylococcus epidermidis strains isolated from breast milk of women suffering infectious mastitis: Potential virulence traits and resistance to antibiotics. BMC Microbiol. 9 (1), 1–11. doi: 10.1186/1471-2180-9-82 19422689 PMC2685400

[B39] Delorme C. Abraham A.-L. Renault P. Guédon E. (2015). Genomics of streptococcus salivarius, a major human commensal. Infect. Genet. Evol. 33, 381–392. doi: 10.1016/j.meegid.2014.10.001 25311532

[B40] Diepers A.-C. Krömker V. Zinke C. Wente N. Pan L. Paulsen K.. (2017). *In vitro* ability of lactic acid bacteria to inhibit mastitis-causing pathogens. Sustain. Chem. Pharm. 5, 84–92. doi: 10.1016/j.scp.2016.06.002

[B41] Do T. Jolley K. A. Maiden M. C. Gilbert S. C. Clark D. Wade W. G.. (2009). Population structure of streptococcus oralis. Microbiology 155 (Pt 8), 2593–2602. doi: 10.1099/mic.0.027284-0 19423627 PMC2885674

[B42] Dror D. K. Allen L. H. (2018). Overview of nutrients in human milk. Adv. Nutr. 9 (suppl_1), 278S–294S. doi: 10.1093/advances/nmy022 29846526 PMC6008960

[B43] Erick M. (2018). Breast milk is conditionally perfect. Med. Hypotheses 111, 82–89. doi: 10.1016/j.mehy.2017.12.020 29407004

[B44] Fedler K. A. Biedenbach D. J. Jones R. N. (2006). Assessment of pathogen frequency and resistance patterns among pediatric patient isolates: Report from the 2004 SENTRY antimicrobial surveillance program on 3 continents. Diagn. Microbiol. Infect. Dis. 56 (4), 427–436. doi: 10.1016/j.diagmicrobio.2006.07.003 16938419

[B45] Fernández L. Langa S. Martín V. Maldonado A. Jiménez E. Martín R.. (2013). The human milk microbiota: Origin and potential roles in health and disease. Pharmacol. Res. 69 (1), 1–10. doi: 10.1016/j.phrs.2012.09.001 22974824

[B46] Fitzstevens J. L. Smith K. C. Hagadorn J. I. Caimano M. J. Matson A. P. Brownell E. A. (2017). Systematic review of the human milk microbiota. Nutr. Clin. Pract. 32 (3), 354–364. doi: 10.1177/0884533616670150 27679525

[B47] Fluit A. C. Rogers M. R. C. Díez-Aguilar M. Cantón R. Benaissa-Trouw B. J. Bayjanov J. R.. (2020). Draft genome sequence of the strain 16-537536, isolated from a patient with bronchiectasis and its relationship to the pseudomonas koreensis group of the pseudomonas fluorescens complex. BMC Res. Notes 13 (1), 10. doi: 10.1186/s13104-019-4863-2 31907003 PMC6945793

[B48] Fudou R. Jojima Y. Seto A. Yamada K. Kimura E. Nakamatsu T.. (2002). Corynebacterium efficiens sp. nov., a glutamic-acid-producing species from soil and vegetables. Int. J. syst. evol. Microbiol. 52 (4), 1127–1131. doi: 10.1099/00207713-52-4-1127 12148616

[B49] Furushita M. Okamoto A. Maeda T. Ohta M. Shiba T. (2005). Isolation of multidrug-resistant stenotrophomonas maltophilia from cultured yellowtail (Seriola quinqueradiata) from a marine fish farm. Appl. Environ. Microbiol. 71 (9), 5598–5600. doi: 10.1128/AEM.71.9.5598-5600.2005 16151156 PMC1214673

[B50] Gao Z. Daliri E. B.-M. Wang J. Liu D. Chen S. Ye X.. (2019). Inhibitory effect of lactic acid bacteria on foodborne pathogens: A review. J. Food Prot. 82 (3), 441–453. doi: 10.4315/0362-028X.JFP-18-303 30794461

[B51] Garrido-Sanz D. Meier-Kolthoff J. P. Göker M. Martin M. Rivilla R. Redondo-Nieto M. (2016). Genomic and genetic diversity within the pseudomonas fluorescens complex. PloS One 11 (2), e0150183. doi: 10.1371/journal.pone.0150183 26915094 PMC4767706

[B52] Garriss G. Nannapaneni P. Simões A. S. Browall S. Subramanian K. Sá-Leão R.. (2019). Genomic characterization of the emerging pathogen streptococcus pseudopneumoniae. MBio 10 (3), e01286–e01219. doi: 10.1128/mBio.01286-19 31239383 PMC6593409

[B53] Geddes D. T. Gridneva Z. Perrella S. L. Mitoulas L. R. Kent J. C. Stinson L. F.. (2021). 25 years of research in human lactation: From discovery to translation. Nutrients 13 (9), 3071, 1–46. doi: 10.3390/nu13093071 PMC8465002 34578947

[B54] George A. D. Gay M. C. Murray K. Muhlhausler B. S. Wlodek M. E. Geddes D. T. (2020). Human milk sampling protocols affect estimation of infant lipid intake. J. Nutr. 150 (11), 2924–2930. doi: 10.1093/jn/nxaa246 32886106 PMC7675139

[B55] Gomila M. Peña A. Mulet M. Lalucat J. García-Valdés E. (2015). Phylogenomics and systematics in pseudomonas. Front. Microbiol. 6, 214. doi: 10.3389/fmicb.2015.00214 26074881 PMC4447124

[B56] Gonzalez E. Brereton N. J. Li C. Lopez Leyva L. Solomons N. W. Agellon L. B.. (2021). Distinct changes occur in the human breast milk microbiome between early and established lactation in breastfeeding Guatemalan mothers. Front. Microbiol. 12. doi: 10.3389/fmicb.2021.557180 PMC7907006 33643228

[B57] Gonzalez E. Pitre F. E. Brereton N. J. (2019). ANCHOR: A 16S rRNA gene amplicon pipeline for microbial analysis of multiple environmental samples. Environ. Microbiol. 21 (7), 2440–2468. doi: 10.1111/1462-2920.14632 30990927 PMC6851558

[B58] Gough E. K. Stephens D. A. Moodie E. E. Prendergast A. J. Stoltzfus R. J. Humphrey J. H.. (2015). Linear growth faltering in infants is associated with acidaminococcus sp. and community-level changes in the gut microbiota. Microbiome 3 (1), 1–10. doi: 10.1186/s40168-015-0089-2 26106478 PMC4477476

[B59] Gridneva Z. Rea A. Tie W. J. Lai C. T. Kugananthan S. Ward L. C.. (2019). Carbohydrates in human milk and body composition of term infants during the first 12 months of lactation. Nutrients 11 (7), 1472. doi: 10.3390/nu11071472 31261649 PMC6683013

[B60] Gröschel M. I. Meehan C. J. Barilar I. Diricks M. Gonzaga A. Steglich M.. (2020). The phylogenetic landscape and nosocomial spread of the multidrug-resistant opportunist stenotrophomonas maltophilia. Nat. Commun. 11 (1), 2044. doi: 10.1038/s41467-020-15123-0 32341346 PMC7184733

[B61] Gross H. Loper J. E. (2009). Genomics of secondary metabolite production by pseudomonas spp. Natural prod. Rep. 26 (11), 1408–1446. doi: 10.1039/b817075b 19844639

[B62] Gutiérrez-Venegas G. Gómez-Mora J. A. Meraz-Rodríguez M. A. Flores-Sánchez M. A. Ortiz-Miranda L. F. (2019). Effect of flavonoids on antimicrobial activity of microorganisms present in dental plaque. Heliyon 5 (12), e03013. doi: 10.1016/j.heliyon.2019.e03013 31886429 PMC6921118

[B63] Haas D. Défago G. (2005). Biological control of soil-borne pathogens by fluorescent pseudomonads. Nat. Rev. Microbiol. 3 (4), 307–319. doi: 10.1038/nrmicro1129 15759041

[B64] Heidari Nonakaran S. Pazhouhandeh M. Keyvani A. Abdollahipour F. Z. Shirzad A. (2015). Isolation and identification of pseudomonas azotoformans for induced calcite precipitation. World J. Microbiol. Biotechnol. 31 (12), 1993–2001. doi: 10.1007/s11274-015-1948-5 26386580

[B65] Heikkilä M. P. Saris P. (2003). Inhibition of staphylococcus aureus by the commensal bacteria of human milk. J. Appl. Microbiol. 95 (3), 471–478. doi: 10.1046/j.1365-2672.2003.02002.x 12911694

[B66] Hunt K. M. Foster J. A. Forney L. J. Schütte U. M. Beck D. L. Abdo Z.. (2011). Characterization of the diversity and temporal stability of bacterial communities in human milk. PloS One 6 (6), e21313. doi: 10.1371/journal.pone.0021313 21695057 PMC3117882

[B67] Hussain N. Li R. Takala T. M. Tariq M. Zaidi A. H. Saris P. E. (2021). Generation of lactose-and protease-positive probiotic lacticaseibacillus rhamnosus GG by conjugation with lactococcus lactis NCDO 712. Appl. Environ. Microbiol. 87 (6), e02957–e02920. doi: 10.1128/AEM.02957-20 33419737 PMC8105022

[B68] Jiménez E. de Andrés J. Manrique M. Pareja-Tobes P. Tobes R. Martínez-Blanch J. F.. (2015). Metagenomic analysis of milk of healthy and mastitis-suffering women. J. Hum. Lactation 31 (3), 406–415. doi: 10.1177/0890334415585078 25948578

[B69] Jost T. Lacroix C. Braegger C. Chassard C. (2013). Assessment of bacterial diversity in breast milk using culture-dependent and culture-independent approaches. Br. J. Nutr. 110 (7), 1253–1262. doi: 10.1017/S0007114513000597 23507238

[B70] Kalathinathan P. Kodiveri Muthukaliannan G. (2021). Characterisation of a potential probiotic strain paracoccus marcusii KGP and its application in whey bioremediation. Folia Microbiol. 66 (5), 819–830. doi: 10.1007/s12223-021-00886-w 34148171

[B71] Kandi V. Palange P. Vaish R. Bhatti A. B. Kale V. Kandi M. R.. (2016). Emerging bacterial infection: Identification and clinical significance of kocuria species. Cureus 8 (8). doi: 10.7759/cureus.731 PMC5017880 27630804

[B72] Kaur M. Jangra M. Singh H. Tambat R. Singh N. Jachak S. M.. (2019). Pseudomonas koreensis recovered from raw yak milk synthesizes a β-carboline derivative with antimicrobial properties. Front. Microbiol. 1728. doi: 10.3389/fmicb.2019.01728 PMC6681700 31417521

[B73] Kilian M. Riley D. R. Jensen A. Brüggemann H. Tettelin H. (2014). Parallel evolution of streptococcus pneumoniae and streptococcus mitis to pathogenic and mutualistic lifestyles. MBio 5 (4), e01490–e01414. doi: 10.1128/mBio.01490-14 25053789 PMC4120201

[B74] Kim S. B. Nedashkovskaya O. I. Mikhailov V. V. Han S. K. Kim K.-O. Rhee M.-S.. (2004). Kocuria marina sp. nov., a novel actinobacterium isolated from marine sediment. Int. J. syst. evol. Microbiol. 54 (5), 1617–1620. doi: 10.1099/ijs.0.02742-0 15388718

[B75] Klijn N. Weerkamp A. H. de Vos W. M. (1995). Genetic marking of lactococcus lactis shows its survival in the human gastrointestinal tract. Appl. Environ. Microbiol. 61 (7), 2771–2774. doi: 10.1128/aem.61.7.2771-2774.1995 7618890 PMC167550

[B76] Klindworth A. Pruesse E. Schweer T. Peplies J. Quast C. Horn M.. (2013). Evaluation of general 16S ribosomal RNA gene PCR primers for classical and next-generation sequencing-based diversity studies. Nucleic Acids Res. 41 (1), e1–e1. doi: 10.1093/nar/gks808 22933715 PMC3592464

[B77] Kloos W. E. Musselwhite M. S. (1975). Distribution and persistence of staphylococcus and micrococcus species and other aerobic bacteria on human skin. Appl. Microbiol. 30 (3), 381–395. doi: 10.1128/am.30.3.381-395.1975 810086 PMC187193

[B78] Ku S. Hsueh P. Yang P. Luh K. (2000). Clinical and microbiological characteristics of bacteremia caused by acinetobacter lwoffii. Eur. J. Clin. Microbiol. Infect. Dis. 19 (7), 501–505. doi: 10.1007/s100960000315 10968320

[B79] Lackey K. A. Williams J. E. Meehan C. L. Zachek J. A. Benda E. D. Price W. J.. (2019). What's normal? microbiomes in human milk and infant feces are related to each other but vary geographically: The INSPIRE study. Front. Nutr. 6. doi: 10.3389/fnut.2019.00045 PMC6479015 31058158

[B80] Lagström H. Rautava S. Ollila H. Kaljonen A. Turta O. Mäkelä J.. (2020). Associations between human milk oligosaccharides and growth in infancy and early childhood. Am. J. Clin. Nutr. 111 (4), 769–778. doi: 10.1093/ajcn/nqaa010 32068776 PMC7138667

[B81] Li C. Solomons N. W. Scott M. E. Koski K. G. (2016). Minerals and trace elements in human breast milk are associated with Guatemalan infant anthropometric outcomes within the first 6 months. J. Nutr. 146 (10), 2067–2074. doi: 10.3945/jn.116.232223 27558578

[B82] Li C. Solomons N. W. Scott M. E. Koski K. G. (2019). Anthropometry before day 46 and growth velocity before 6 months of Guatemalan breastfed infants are associated with subclinical mastitis and milk cytokines, minerals, and trace elements. J. Nutr. 149 (9), 1651–1659. doi: 10.1093/jn/nxz109 31187864

[B83] Lopes L. D. Pereira e Silva M.d.C. Weisberg A. J. Davis E. W. Yan Q. Varize C.d.S.. (2018). Genome variations between rhizosphere and bulk soil ecotypes of a pseudomonas koreensis population. Environ. Microbiol. 20 (12), 4401–4414. doi: 10.1111/1462-2920.14363 30033663

[B84] Love M. I. Huber W. Anders S. (2014). Moderated estimation of fold change and dispersion for RNA-seq data with DESeq2. Genome Biol. 15 (12), 550. doi: 10.1186/s13059-014-0550-8 25516281 PMC4302049

[B85] Lozano G. L. Bravo J. I. Handelsman J. (2017). Draft genome sequence of pseudomonas koreensis CI12, a bacillus cereus “hitchhiker” from the soybean rhizosphere. Genome Announce. 5 (26), e00570–e00517. doi: 10.1128/genomeA.00570-17 PMC5638277 28663293

[B86] Łubiech K. Twarużek M. (2020). Lactobacillus bacteria in breast milk. Nutrients 12 (12), 3783. doi: 10.3390/nu12123783 33321792 PMC7764098

[B87] Luque V. Closa-Monasterolo R. Escribano J. Ferré N. (2015). Early programming by protein intake: The effect of protein on adiposity development and the growth and functionality of vital organs. Nutr. Metab. Insights 8, NMI. S29525. doi: 10.4137/NMI.S29525 PMC4803318 27013888

[B88] Lyons K. E. Ryan C. A. Dempsey E. M. Ross R. P. Stanton C. (2020). Breast milk, a source of beneficial microbes and associated benefits for infant health. Nutrients 12 (4), 1039; 1–30. doi: 10.3390/nu12041039 PMC7231147 32283875

[B89] Maidak B. L. Cole J. R. Lilburn T. G. Parker C. T. Jr. Saxman P. R. Stredwick J. M.. (2000). The RDP (ribosomal database project) continues. Nucleic Acids Res. 28 (1), 173–174. doi: 10.1093/nar/28.1.173 10592216 PMC102428

[B90] Mantaring J. Benyacoub J. Destura R. Pecquet S. Vidal K. Volger S.. (2018). Effect of maternal supplement beverage with and without probiotics during pregnancy and lactation on maternal and infant health: A randomized controlled trial in the Philippines. BMC Pregnancy Childbirth 18 (1), 193. doi: 10.1186/s12884-018-1828-8 29855271 PMC5984298

[B91] Martín R. Heilig H. G. Zoetendal E. G. Jiménez E. Fernández L. Smidt H.. (2007). Cultivation-independent assessment of the bacterial diversity of breast milk among healthy women. Res. Microbiol. 158 (1), 31–37. doi: 10.1016/j.resmic.2006.11.004 17224259

[B92] Matsumoto T. Cifuentes O. Masunaga T. (2013). Characterization of soil properties in relation to maize productivity in andosols of the western highland of Guatemala. Soil Sci. Plant Nutr. 59 (2), 195–207. doi: 10.1080/00380768.2012.760430

[B93] Mauchline T. H. Malone J. G. (2017). Life in earth – the root microbiome to the rescue? Curr. Opin. Microbiol. 37, 23–28. doi: 10.1016/j.mib.2017.03.005 28437662

[B94] Mediano P. Fernández L. Jiménez E. Arroyo R. Espinosa-Martos I. Rodríguez J. M.. (2017). Microbial diversity in milk of women with mastitis: potential role of coagulase-negative staphylococci, viridans group streptococci, and corynebacteria. J. Hum. Lactation 33 (2), 309–318. doi: 10.1177/0890334417692968 28418794

[B95] McMurdie P. J. Holmes S. (2013). Phyloseq: An R package for reproducible interactive analysis and graphics of microbiome census data. PloS One 8 (4), e61217. doi: 10.1371/journal.pone.0061217 23630581 PMC3632530

[B96] Mitchell J. (2011). Streptococcus mitis: Walking the line between commensalism and pathogenesis. Mol. Oral. Microbiol. 26 (2), 89–98. doi: 10.1111/j.2041-1014.2010.00601.x 21375700

[B97] Monira S. Nakamura S. Gotoh K. Izutsu K. Watanabe H. Alam N.H. (2011). Gut microbiota of healthy and malnourished children in Bangladesh. Frontiers in microbiology 2, 228. doi: 10.3389/fmicb.2011.00228 22125551 PMC3221396

[B98] Naidu A. (2000). Natural food antimicrobial systems (Boca Raton, FL: CRC press).

[B99] Neumann C. G. Oace S. M. Chaparro M. P. Herman D. Drorbaugh N. Bwibo N. O. (2013). Low vitamin B12 intake during pregnancy and lactation and low breastmilk vitamin 12 content in rural Kenyan women consuming predominantly maize diets. Food Nutr. Bull. 34 (2), 151–159. doi: 10.1177/156482651303400204 23964388

[B100] O'Leary N. A. Wright M. W. Brister J. R. Ciufo S. Haddad D. McVeigh R.. (2016). Reference sequence (RefSeq) database at NCBI: Current status, taxonomic expansion, and functional annotation. Nucleic Acids Res. 44 (D1), D733–D745. doi: 10.1093/nar/gkv1189 26553804 PMC4702849

[B101] Otto M. (2009). Staphylococcus epidermidis–the'accidental'pathogen. Nat. Rev. Microbiol. 7 (8), 555–567. doi: 10.1038/nrmicro2182 19609257 PMC2807625

[B102] Pannaraj P. S. Li F. Cerini C. Bender J. M. Yang S. Rollie A.. (2017). Association between breast milk bacterial communities and establishment and development of the infant gut microbiome. JAMA Pediatr. 171 (7), 647–654. doi: 10.1001/jamapediatrics.2017.0378 28492938 PMC5710346

[B103] Pastor-Villaescusa B. Hurtado J. Gil-Campos M. Uberos J. Maldonado-Lobón J. Díaz-Ropero M.. (2020). Effects of lactobacillus fermentum CECT5716 Lc40 on infant growth and health: A randomised clinical trial in nursing women. Beneficial Microbes 11 (3), 235–244. doi: 10.3920/BM2019.0180 32216468

[B104] Patel S. K. Pratap C. B. Verma A. K. Jain A. K. Dixit V. K. Nath G. (2013). Pseudomonas fluorescens-like bacteria from the stomach: A microbiological and molecular study. World J. Gastroenterol.: WJG 19 (7), 1056–1067. doi: 10.3748/wjg.v19.i7.1056 PMC3581993 23466902

[B105] Patel S. K. Pratap C. B. Verma A. K. Jain A. K. Dixit V. K. Nath G. (2013). Pseudomonas fluorescens-like bacteria from the stomach: A microbiological and molecular study. World J. Gastroenterol. 19 (7), 1056–1067. doi: 10.3748/wjg.v19.i7.1056 23466902 PMC3581993

[B106] Pearce C. Bowden G. Evans M. Fitzsimmons S. Johnson J. Sheridan M.. (1995). Identification of pioneer viridans streptococci in the oral cavity of human neonates. J. Med. Microbiol. 42 (1), 67–72. doi: 10.1099/00222615-42-1-67 7739028

[B107] Perrella S. Gridneva Z. Lai C. T. Stinson L. George A. Bilston-John S.. (2021). Human milk composition promotes optimal infant growth, development and health. Semin. perinatology 45 (2), 151380. doi: 10.1016/j.semperi.2020.151380 33431112

[B108] Perrella S. Gridneva Z. Lai C. T. Stinson L. George A. Bilston-John S.. (2021). “Human milk composition promotes optimal infant growth, development and health,” in Seminars in perinatology (Elsevier), 151380. 10.1016/j.semperi.2020.151380 33431112

[B109] Pinski A. Zur J. Hasterok R. Hupert-Kocurek K. (2020). Comparative genomics of stenotrophomonas maltophilia and stenotrophomonas rhizophila revealed characteristic features of both species. Int. J. Mol. Sci. 21 (14), 4922, 1–20. doi: 10.3390/ijms21144922 32664682 PMC7404187

[B110] Pruesse E. Quast C. Knittel K. Fuchs B. M. Ludwig W. Peplies J.. (2007). SILVA: A comprehensive online resource for quality checked and aligned ribosomal RNA sequence data compatible with ARB. Nucleic Acids Res. 35 (21), 7188–7196. doi: 10.1093/nar/gkm864 17947321 PMC2175337

[B111] Qi H. Liu D. Zou Y. Wang N. Tian H. Xiao C. (2021). Description and genomic characterization of streptococcus symci sp. nov., isolated from a child’s oropharynx. Antonie van Leeuwenhoek 114 (2), 113–127. doi: 10.1007/s10482-020-01505-3 33387140 PMC7878260

[B112] Ramani S. Stewart C. J. Laucirica D. R. Ajami N. J. Robertson B. Autran C. A.. (2018). Human milk oligosaccharides, milk microbiome and infant gut microbiome modulate neonatal rotavirus infection. Nat. Commun. 9 (1), 1–12. doi: 10.1038/s41467-018-07476-4 30479342 PMC6258677

[B113] Ramsay D. T. Kent J. C. Owens R. A. Hartmann P. E. (2004). Ultrasound imaging of milk ejection in the breast of lactating women. Pediatrics 113 (2), 361–367. doi: 10.1542/peds.113.2.361 14754950

[B114] Rao M. R. K. Kumar S. S. (2014). Identification of two new bacterial species, brevundimonas nasdae and microbacterium trichothecenolyticum from kolavai lake, chengalpattu, Tamil nadu, India. Am. J. PharmTech. Res. 4 (5), 736–751.

[B115] Reichmann P. Nuhn M. Denapaite D. Brückner R. Henrich B. Maurer P.. (2011). "Genome of streptococcus oralis strain Uo5". Am. Soc. Microbiol 193 (11), 2888–2889. doi: 10.1128/JB.00321-11 PMC3133139 21460080

[B116] Ren H. Du M. (2017). Role of maternal periodontitis in preterm birth. Front. Immunol. 8, 139. doi: 10.3389/fimmu.2017.00139 28243243 PMC5303728

[B117] Ren Y. Yao M. Chang P. Sun Y. Li R. Meng D.. (2021). Isolation and characterization of a pseudomonas poae JSU-Y1 with patulin degradation ability and biocontrol potential against penicillium expansum. Toxicon 195, 1–6. doi: 10.1016/j.toxicon.2021.02.014 33640407

[B118] Rivera J. Ruel M. T. (1997). Growth retardation starts in the first three months of life among rural Guatemalan children. Eur. J. Clin. Nutr. 51 (2), 92–96. doi: 10.1038/sj.ejcn.1600371 9049567

[B119] Robertson R. C. Manges A. R. Finlay B. B. Prendergast A. J. (2019). The human microbiome and child growth–first 1000 days and beyond. Trends Microbiol. 27 (2), 131–147. doi: 10.1016/j.tim.2018.09.008 30529020

[B120] Ruel M. T. Rivera J. Habicht J.-P. (1995). Length screens better than weight in stunted populations. J. Nutr. 125 (5), 1222–1228. 7738682 10.1093/jn/125.5.1222

[B121] Ryan M. P. Pembroke J. T. (2018). Brevundimonas spp: Emerging global opportunistic pathogens. Virulence 9 (1), 480–493. doi: 10.1080/21505594.2017.1419116 29484917 PMC5955483

[B122] Scales B. S. Dickson R. P. LiPuma J. J. Huffnagle G. B. (2014). Microbiology, genomics, and clinical significance of the pseudomonas fluorescens species complex, an unappreciated colonizer of humans. Clin. Microbiol. Rev. 27 (4), 927–948. doi: 10.1128/CMR.00044-14 25278578 PMC4187640

[B123] Schloss P. D. Westcott S. L. Ryabin T. Hall J. R. Hartmann M. Hollister E. B.. (2009). Introducing mothur: Open-source, platform-independent, community-supported software for describing and comparing microbial communities. Appl. Environ. Microbiol. 75 (23), 7537–7541. doi: 10.1128/AEM.01541-09 19801464 PMC2786419

[B124] Schmidt C. S. Alavi M. Cardinale M. Müller H. Berg G. (2012). Stenotrophomonas rhizophila DSM14405T promotes plant growth probably by altering fungal communities in the rhizosphere. Biol. Fertil. Soils 48 (8), 947–960. doi: 10.1007/s00374-012-0688-z

[B125] Schwarzer M. Makki K. Storelli G. Machuca-Gayet I. Srutkova D. Hermanova P.. (2016). Lactobacillus plantarum strain maintains growth of infant mice during chronic undernutrition. Science 351 (6275), 854–857. doi: 10.1126/science.aad8588 26912894

[B126] Schwarzer M. Poinsot P. Lambert A. Goeffroy S. Peretti N. Leulier F. (2018). A310 daily administration of latobacillus plantarum imptoves mouse juvenile growth kinetics by sustaining somatotropic axis activity upon undernutrition. J. Can. Assoc. Gastroenterol. 1 (suppl_2), 445–445. doi: 10.1093/jcag/gwy009.310

[B127] Sharma C. Singh B. P. Thakur N. Gulati S. Gupta S. Mishra S. K.. (2017). Antibacterial effects of lactobacillus isolates of curd and human milk origin against food-borne and human pathogens. 3 Biotech. 7 (1), 31. doi: 10.1007/s13205-016-0591-7 PMC5388649 28401466

[B128] Shaw L. Harjunmaa U. Doyle R. Mulewa S. Charlie D. Maleta K.. (2016). Distinguishing the signals of gingivitis and periodontitis in supragingival plaque: A cross-sectional cohort study in Malawi. Appl. Environ. Microbiol. 82 (19), 6057–6067. doi: 10.1128/AEM.01756-16 27520811 PMC5038043

[B129] Shin N.-R. Whon T. W. Bae J.-W. (2015). Proteobacteria: microbial signature of dysbiosis in gut microbiota. Trends Biotechnol. 33 (9), 496–503. doi: 0.1016/j.tibtech.2015.06.011 26210164 10.1016/j.tibtech.2015.06.011

[B130] Silambarasan S. Logeswari P. Ruiz A. Cornejo P. Kannan V. R. (2020). Influence of plant beneficial stenotrophomonas rhizophila strain CASB3 on the degradation of diuron-contaminated saline soil and improvement of lactuca sativa growth. Environ. Sci. pollut. Res. 27 (28), 35195–35207. doi: 10.1007/s11356-020-09722-z 32588300

[B131] Silby M. W. Cerdeño-Tárraga A. M. Vernikos G. S. Giddens S. R. Jackson R. W. Preston G. M.. (2009). Genomic and genetic analyses of diversity and plant interactions of pseudomonas fluorescens. Genome Biol. 10 (5), 1–16. doi: 10.1186/gb-2009-10-5-r51 PMC2718517 19432983

[B132] Siqueira F. M. Cota L. O. M. Costa J. E. Haddad J. P. A. Lana Â.M.Q. Costa F. O. (2007). Intrauterine growth restriction, low birth weight, and preterm birth: adverse pregnancy outcomes and their association with maternal periodontitis. J. periodontol. 78 (12), 2266–2276. doi: 10.1902/jop.2007.070196 18052698

[B133] Smeets J. Löwe S. Veraart J. (2007). Cutaneous infections with stenotrophomonas maltophilia in patients using immunosuppressive medication. J. Eur. Acad. Dermatol. Venereol. 21 (9), 1298–1300. doi: 10.1111/j.1468-3083.2007.02201.x 17894750

[B134] Soeorg H. Huik K. Parm Ü. Ilmoja M.-L. Metelskaja N. Metsvaht T.. (2013). Genetic relatedness of coagulase-negative staphylococci from gastrointestinal tract and blood of preterm neonates with late-onset sepsis. Pediatr. Infect. Dis. J. 32 (4), 389–393. doi: 10.1097/INF.0b013e3182791abd 23080292

[B135] Soeorg H. Metsvaht T. Eelmäe I. Merila M. Treumuth S. Huik K.. (2017). The role of breast milk in the colonization of neonatal gut and skin with coagulase-negative staphylococci. Pediatr. Res. 82 (5), 759–767. doi: 10.1038/pr.2017.150 28665928

[B136] Solomons N. W. Vossenaar M. Chomat A.-M. Doak C. M. Koski K. G. Scott M. E. (2015). Stunting at birth: Recognition of early-life linear growth failure in the western highlands of Guatemala. Public Health Nutr. 18 (10), 1737–1745. doi: 10.1017/S136898001400264X 26017476 PMC10271386

[B137] Soltani S. Hammami R. Cotter P. D. Rebuffat S. Said L. B. Gaudreau H.. (2020). Bacteriocins as a new generation of antimicrobials: Toxicity aspects and regulations. FEMS Microbiol. Rev. 45 (1), 1–24. doi: 10.1093/femsre/fuaa039 PMC7794045 32876664

[B138] Spencer R. (1995). The emergence of epidemic, multiple-antibiotic-resistant stenotrophomonas (Xanthomonas) maltophilia and burkholderia (Pseudomonas) cepacia. J. Hosp. Infect. 30, 453–464. doi: 10.1016/0195-6701(95)90049-7 7560984

[B139] Srinivas S. K. Sammel M. D. Stamilio D. M. Clothier B. Jeffcoat M. K. Parry S.. (2009). Periodontal disease and adverse pregnancy outcomes: Is there an association? Am. J. obstetr. gynecol. 200 (5), 497. e491–497. e498. doi: 10.1016/j.ajog.2009.03.003 19375568

[B140] Stabler S. N. Mack B. McCormack G. Cheng M. P. (2018). Brevundimonas vesicularis causing bilateral pneumosepsis in an immunocompetent adult: A case report and literature review. Can. J. Hosp. Pharm. 71 (3), 208. doi: 10.4212/cjhp.v71i3.2587 29955194 PMC6019083

[B141] Stackebrandt E. Koch C. Gvozdiak O. Schumann P. (1995). Taxonomic dissection of the genus micrococcus: Kocuria gen. nov., nesterenkonia gen. nov., kytococcus gen. nov., dermacoccus gen. nov., and micrococcus Cohn 1872 gen. emend. Int. J. Syst. Bacteriol 45 (4), 682–692. doi: 10.1099/00207713-45-4-682 7547287

[B142] Stevens G. A. Finucane M. M. Paciorek C. J. Flaxman S. R. White R. A. Donner A. J.. (2012). Trends in mild, moderate, and severe stunting and underweight, and progress towards MDG 1 in 141 developing countries: A systematic analysis of population representative data. Lancet 380 (9844), 824–834. doi: 10.1016/S0140-6736(12)60647-3 22770478 PMC3443900

[B143] Stewart C. J. Ajami N. J. O’Brien J. L. Hutchinson D. S. Smith D. P. Wong M. C.. (2018). Temporal development of the gut microbiome in early childhood from the TEDDY study. Nature 562 (7728), 583–588. doi: 10.1038/s41586-018-0617-x 30356187 PMC6415775

[B144] Stinson L. F. Sindi A. S. Cheema A. S. Lai C. T. Mühlhäusler B. S. Wlodek M. E.. (2021). The human milk microbiome: Who, what, when, where, why, and how? Nutr. Rev. 79 (5), 529–543. doi: 10.1093/nutrit/nuaa029 32443154

[B145] Tasnim N. Abulizi N. Pither J. Hart M. M. Gibson D. L. (2017). Linking the gut microbial ecosystem with the environment: Does gut health depend on where we live? Front. Microbiol. 8, 1935. doi: 10.3389/fmicb.2017.01935 29056933 PMC5635058

[B146] Togo A. Dufour J. C. Lagier J. C. Dubourg G. Raoult D. Million M. (2019). Repertoire of human breast and milk microbiota: A systematic review. Future Microbiol. 14, 623–641. doi: 10.2217/fmb-2018-0317 31025880

[B147] Trucksess M. W. Diaz-Amigo C. (2011). “Mycotoxins in foods,” in Encyclopedia of environmental health. Ed. Nriagu J. O. (Burlington: Elsevier), 888–897.

[B148] Turroni F. Milani C. Duranti S. Ferrario C. Lugli G. A. Mancabelli L.. (2018). Bifidobacteria and the infant gut: An example of co-evolution and natural selection. Cell. Mol. Life Sci. 75 (1), 103–118. doi: 10.1007/s00018-017-2672-0 28983638 PMC11105234

[B149] Urakami T. Araki H. Oyanagi H. Suzuki K.-I. Komagata K. (1990). Paracoccus aminophilus sp. nov. and paracoccus aminovorans sp. nov., which utilize n, n-dimethylformamide. Int. J. Syst. Evol. Microbiol. 40 (3), 287–291. doi: 10.1099/00207713-40-3-287 2397196

[B150] Volke D. C. Calero P. Nikel P. I. (2020). Pseudomonas putida. Trends Microbiol. 28 (512-513), 1. doi: 10.1016/j.tim.2020.02.015 32396829

[B151] Wiciński M. Sawicka E. Gębalski J. Kubiak K. Malinowski B. (2020). Human milk oligosaccharides: Health benefits, potential applications in infant formulas, and pharmacology. Nutrients 12 (1), 266; 1–14. doi: 10.3390/nu12010266 PMC7019891 31968617

[B152] Wolf A. Fritze A. Hagemann M. Berg G. (2002). Stenotrophomonas rhizophila sp. nov., a novel plant-associated bacterium with antifungal properties. Int. J. syst. evol. Microbiol. 52 (6), 1937–1944. doi: 10.1099/00207713-52-6-1937 12508851

[B153] World Bank (2021) The world bank in Guatemala. Available at: https://www.wfp.org/countries/guatemala

[B154] World Health Organization (2006). WHO child growth standards: length/height-for-age, weight-for-age, weight-for-length, weight-for-height and body mass index-for-age: methods and development. Available at: https://www.who.int/publications/i/item/924154693X.

[B155] World Health Organization (2017). Guideline: Protecting, promoting and supporting breastfeeding in facilities providing maternity and newborn services. Available at: https://www.who.int/publications/i/item/9789241550086. 29565522

[B156] Wren H. M. Solomons N. W. Chomat A. M. Scott M. E. Koski K. G. (2015). Cultural determinants of optimal breastfeeding practices among indigenous mam-Mayan women in the Western highlands of Guatemala. J. Hum. Lactation 31 (1), 172–184. doi: 10.1177/0890334414560194 25583316

[B157] Xue J. Shen K. Hu Y. Hu Y. Kumar V. Yang G.. (2020). Effects of dietary bacillus cereus, b. subtilis, paracoccus marcusii, and lactobacillus plantarum supplementation on the growth, immune response, antioxidant capacity, and intestinal health of juvenile grass carp (Ctenopharyngodon idellus). Aquac. Rep. 17, 100387. doi: 10.1016/j.aqrep.2020.100387

[B158] Yang G. Tian X. Dong S. Peng M. Wang D. (2015). Effects of dietary bacillus cereus G19, b. cereus BC-01, and paracoccus marcusii DB11 supplementation on the growth, immune response, and expression of immune-related genes in coelomocytes and intestine of the sea cucumber (Apostichopus japonicus selenka). Fish shellfish Immunol. 45 (2), 800–807. doi: 10.1016/j.fsi.2015.05.032 26052012

[B159] Yoon E.-J. Goussard S. Touchon M. Krizova L. Cerqueira G. Murphy C.. (2014). Origin in acinetobacter guillouiae and dissemination of the aminoglycoside-modifying enzyme aph (3′)-VI. MBio 5 (5), e01972–e01914. doi: 10.1128/mBio.01972-14 25336457 PMC4212838

[B160] Young B. E. Patinkin Z. W. Pyle L. de la Houssaye B. Davidson B. S. Geraghty S.. (2017). Markers of oxidative stress in human milk do not differ by maternal BMI but are related to infant growth trajectories. Maternal Child Health J. 21 (6), 1367–1376. doi: 10.1007/s10995-016-2243-2 PMC5444997 28138825

